# The roles of expectation, comparator, administration route, and population in open-label placebo effects: a network meta-analysis

**DOI:** 10.1038/s41598-023-39123-4

**Published:** 2023-07-22

**Authors:** Sarah Buergler, Dilan Sezer, Jens Gaab, Cosima Locher

**Affiliations:** 1 Division of Clinical Psychology and Psychotherapy, Faculty of Psychology, University of Basel, Basel, Switzerland; 2 Department of Consultation-Liaison Psychiatry and Psychosomatic Medicine, University Hospital Zurich, University of Zurich, Zurich, Switzerland; 3 Faculty of Health, University of Plymouth, Plymouth, UK

**Keywords:** Psychology, Medical ethics

## Abstract

Three meta-analyses have demonstrated the clinical potential of open-label placebos (OLPs). However, there is a need to synthesize the existing evidence through more complex analyses that would make it possible to answer questions beyond mere efficacy. Such analyses would serve to improve the understanding of why and under what circumstances OLPs work (e.g., depending on induced expectations or across different control groups). To answer these questions, we conducted the first network meta-analyses in the field of OLPs. Our analyses revealed that OLPs could be beneficial in comparison to no treatment in nonclinical (12 trials; 1015 participants) and clinical populations (25 trials; 2006 participants). Positive treatment expectations were found to be important for OLPs to work. Also, OLP effects can vary depending on the comparator used. While the kind of administration route had no substantial impact on the OLP effects, effects were found to be larger in clinical populations than in nonclinical populations. These results suggest that the expectation, comparator, administration route, and population should be considered when designing and interpreting OLP studies.

## Introduction

Placebos have been found to have clinically significant effects in a variety of clinical conditions^1,2^, but they are not allowed to be used in clinical practice because their use would violate ethical obligations. In this regard, open-label placebos (OLPs) administered under full disclosure and transparency can be considered both ethical and feasible^3^. Several meta-analyses have shown medium-sized to large clinically relevant effects of OLPs on self-reported outcomes^4–6^ that can be comparable in magnitude to deceptively administered placebos (DPs)^7–12^. However, given that this field of research is still in its infancy—the first controlled study was published in 2008^13^—there are still many questions that need to be addressed.

The associative learning (i.e., conditioning) and verbal suggestions that accompany a treatment play a key role in the expected and actual placebo effects^14^. In the majority of OLP studies, the distribution of the OLP is accompanied by a rationale consisting of four discussion points in order to induce positive treatment expectations (see, e.g., Ref.^15^). So far, the impact of positive expectation on OLP effects has been explored in studies comparing OLPs with expectation induction (i.e., through verbal suggestions or conditioning) to OLPs without such expectation-inducing procedures (hereafter, OLP −)^8,16–19^. Some authors have concluded that the treatment rationale is crucial when it comes to the efficacy of OLPs (e.g., Ref.^8^), however, systematic investigations have been limited to the experimental context^6^.

Effect sizes may also depend on the different control conditions used in trials. For example, it has been found that wait-list (WL) control groups lead to larger effects than no treatment (NT) controls^20^. This result could be due to the fact that subjects assigned to a WL group are not actively looking for improvement opportunities during the waiting phase, as might be the case with NT groups. Blease et al.^21^ compared this phenomenon with the induction of nocebo effects in the context of OLPs, especially in cases when the experimenter mentions the potential advantages of the OLP intervention before assignment to the WL. Further, the use of treatment as usual (TAU) controls can be considered problematic as the “treatment as usual” is typically not monitored or sufficiently reported, which may lead to structural inequivalence across studies applying TAU^22,23^. It is thus warranted to take a closer look at the different comparators used across OLP studies and their impact on effects.

OLP effects may vary not only depending on induced expectations or across control groups but also across treatment administration routes. For example, more invasive placebo procedures, such as injections and sham procedures, have been shown to increase expectations of a treatment’s efficacy—and in turn, to enhance placebo effects^24–26^. However, while placebo effects in itch seem not to differ between oral and injective placebo administration^27^, intra-articular and topical placebos have been found to be more efficacious than orally administered placebo for osteoarthritis^28^. It has been argued that more invasive administrations of placebos have stronger effects than less invasive administration routes (oral or nasal) in the case of pain, whereas in nonpain conditions, such as itches, this might not be the case^29^. Nonetheless, placebo experts strongly agree that clinicians should not prescribe more invasive treatments merely to obtain stronger placebo effects, due to practical and ethical restrictions, higher costs, and higher risks of undesirable side effects^1^. This is especially true for OLPs, as it remains unclear whether the findings that more invasive treatments are more beneficial in the case of deceptive placebos can be applied to OLPs.

Furthermore, there has not yet been a meta-analysis on OLP that explored the differential effects across distinct populations (i.e., clinical and nonclinical) within a single study. Whereas OLPs have been significantly more efficacious compared to NT with moderate to large effects (*SMD* = 0.88^4^ and 0.72^5^) in clinical conditions, in nonclinical experimental conditions a moderate effect has been found in OLPs for self-reported outcomes (*SMD* = 0.43), and no significant effect has been observed for objective outcomes (*SMD* = − 0.02^6^). It thus appears that OLP effects differ between clinical and nonclinical samples, a finding known in research on deceptive placebos where placebo analgesia tends to be higher in patients compared to healthy subjects^30,31^.

As illustrated above, currently existing meta-analyses on OLP effects have not addressed several important aspects: (1) No review study has systematically examined, on the basis of a relatively large database whether the effects of OLPs with positive expectations, either through a rationale or other expectation-inducing measures (e.g., conditioning), differ from those without expectation induction. (2) Current OLP meta-analyses have lumped together all control groups, so they do not differentiate between different control conditions. Investigating OLP efficacy in comparison to different kinds of control groups is, however, of great importance, as it can ensure that OLP effect sizes are not over- or underestimated. (3) Furthermore, these pairwise meta-analyses have not been able to comprehensively examine the different routes of administration. Hence, it remains unclear whether different placebo administrations result in different effects and whether there is justification for choosing one route of administration over another. (4) Finally, each of the three meta-analyses only considered one type of population (clinical or nonclinical). The comparability of the effect sizes across the different individual meta-analyses might be limited, however, as these studies used different definitions of eligible OLP interventions and of conditions that qualify as nonclinical and clinical populations. This especially holds true for the question whether subclinical conditions (e.g., menopausal hot flushes, self-reported test anxiety, or general well-being) are to be considered nonclinical or clinical. There is therefore a need for a clear definition of these samples and for meta-analytic analyses that apply the same inclusion criteria in both areas.

To examine these open questions, network meta-analysis (NMA) is the method of choice. NMAs allow the comparison of multiple treatment and comparator groups. Further, NMAs produce more accurate effect sizes than a traditional meta-analyses by including both direct and indirect evidence. To the best of our knowledge, this is the first NMA on OLP treatments. On the basis of the challenges discussed above and open questions in OLP research, we derived the following research question (RQ) that can be answered in a network meta-analytic framework: Is the magnitude of the OLP effects different across (RQ1) treatment expectations, (RQ2) comparator groups, (RQ3) OLP treatment administration routes, or (RQ4) clinical vs. nonclinical populations.

## Methods

### Search strategy

A systematic review and NMA was conducted in accordance with the Preferred Reporting Items for Systematic Reviews and Meta-analyses (PRISMA) Statement^32^ (see eAppendix 5 in the supplement). The search strategies were conducted in Medline, Embase, and PsycINFO via Ovid, the Cumulative Index of Nursing and Allied Health Literature (CINAHL), clinicaltrials.gov, OpenTrials, and the Cochrane Register of Controlled Trials and were developed in close collaboration with an information specialist. The four databases and the three registries were searched using text word synonyms and database-specific subject headings for open-label placebos on February 2, 2021 (see eAppendix 1 in the supplement), and updated on June 8, 2022 (see eAppendix 2 in the supplement). No language restrictions were applied. For Medline and Embase, randomized controlled trial (RCT) filters were applied, and conference abstracts and conference reviews were excluded from Embase. References were exported to Endnote X9 and deduplicated using the Bramer method^33^. Furthermore, additional trials were identified from an existing systematic review on OLPs^6^ and a newsletter on placebo studies (https://jips.online/). If data were not available, the corresponding authors of the respective publication were contacted via email. Several reviewers, in pairs of two, independently screened the references based on their titles and abstracts using https://covidence.org. The full texts of selected references were retrieved and independently assessed for eligibility by two reviewers. Any disagreements were resolved by a third reviewer. This study was registered with Prospero (CRD42020161696).

### Study selection

We included RCTs that compared OLPs to a control group in clinical (e.g., chronic low-back pain, depression, irritable bowel syndrome, allergic rhinitis), subclinical (e.g., menopausal hot flushes or test anxiety), and nonclinical (e.g., experimentally induced pain or allergic reactions) populations. There were no age restrictions. Our definition of OLP was as follows: (1) The placebo must have been given openly; that is, the participant must have been 100% aware of receiving a placebo when it was applied. Studies needed to state explicitly that the placebo was delivered with the full awareness of the receiver, so simply using the term *open label* as a description of the study was not sufficient, as this term has been used inconsistently and sometimes indicates that the treatment provider was unblinded. Also, balanced-placebo-design studies (with, e.g., a 50% chance of receiving a placebo) were excluded. (2) The placebo had to consist of a “pharmacological” property, meaning that it could be swallowed (e.g., pills, capsules, syrups, etc.), applied to the skin or other body parts (e.g., creams or eye drops), or injected. Studies that tested placebo devices (e.g., deep brain stimulation), exercises, or diets were excluded. Studies that tested procedures such as placebo massage or acupuncture without including an additional treatment arm that fulfilled our definition of placebos were not eligible. (3) At least a minimal positive expectation needed to be induced alongside the placebo administration (e.g., through a rationale with positive suggestions or conditioning). (4) The placebo needed to be applied with the intention of a positive effect (i.e., of therapeutic effect or of enhancing well-being; no nocebo effects). Based on these criteria, none of the open-label drug trials using OLP as a comparator met our definition, even though we also aimed to include these analyses.

Crossover studies were only included if we were able to extract the results of the first period of the trial (i.e., before the first crossover) separately. This is because data from crossover studies should not be treated as if it stemmed from a parallel trial^34^. If these data were not reported, the authors were contacted. If the authors did not respond or the data could not be obtained for other reasons, the study was excluded from the analysis^13,35–38^. In order to be included, studies needed to report a baseline and a posttreatment measurement or alternatively report change scores from baseline to posttreatment. This is due to the Cochrane Handbook recommendation to only analyze either change or post scores when calculating standardized mean differences (*SMD*s)^34^. In order to decrease between-person variability and thereby increase the efficiency of the analysis, we opted for change scores. Studies reporting only posttreatment values or where we were not able to retrieve means and standard deviations (*SD*s) were therefore excluded^39–43^. For studies published more than once (i.e., secondary analyses), we only included the entry with the most relevant data to our analysis.

### Data extraction

All relevant data were extracted independently in pairs of two using a standardized Excel template. Disagreements were clarified through consensus and by consultation with a third reviewer if required. Means and *SD*s were extracted, and when *SD*s were not reported, we calculated them from standard errors (*SE*), confidence intervals (CIs), or interquartile ranges (IQR); medians were converted to means^34^. If the sample size used for the analysis was not reported, we used the sample size from the baseline data (i.e., participants randomized). If it was not possible to impute the appropriate measures for calculating the effect sizes or if data were missing, we contacted the authors to obtain them. If the authors did not provide the respective information, the study was excluded from further analyses.

### Primary outcomes

We applied a hierarchy for the choice of outcomes: (1) As a first choice, we extracted the primary outcome as defined by the study authors. In the presence of two or more primary outcomes, we checked trial registries for additional information or contacted the authors. If no information could be obtained, the outcome for the present analysis was selected based on (2) the most frequently reported outcome across our data pool (i.e., pain was preferred over medication use) in order to reduce heterogeneity, and if this was not applicable, (3) the most informative outcome (e.g., a symptom-related scale was preferred over a general quality-of-life assessment) was selected. In the absence of a baseline assessment for the primary outcome, another outcome was chosen according to these rules to avoid the exclusion of the study (see eTable 1 in the supplement for the rationale for choosing the outcomes). If more than one baseline measure was collected, we chose the time point closest to the start of the intervention^44^. If more than one posttreatment measurement was reported, we extracted the first assessment after the end of the intervention (i.e., measured at the time point closest to the end of treatment), unless the publication provided an explanation for why another time point was the most clinically relevant (i.e., a definition of the primary endpoint measurement). In studies that included a WL control group, participants additionally received the OLP treatment after the completion of the study. Outcomes for these individuals were not included in the analyses because they lacked a control group for comparison and in order to avoid including participants multiple times.

### Sample building

We allocated each study to either the nonclinical or the clinical study pool. Nonclinical studies were defined as studies with experimentally induced states (i.e., experimentally induced pain, itch, sadness), whereas clinical studies investigated the effects of OLPs in naturally occurring states (e.g., clinical: irritable-bowel syndrome, chronic low-back pain; subclinical: test anxiety, well-being, relaxation). One study^17^ experimentally induced pain in a sample of patients with chronic low-back pain. This study was rubricated as clinical.

### Node building

In order to be able to test the effects of different OLP administration routes in comparison to different control groups, each group in a study was clustered together with similar groups from other studies. Our strategy for creating the nodes was data-based and aimed to restrain the number of nodes. This lumping approach has the methodological advantage of increasing the power and allowing for more accurate estimates of the effect sizes^45,46^. The following rules were applied: (1) Nodes were built according to the OLP administration route, that is, oral pills (capsules, tablets), oral suspensions (drops, solutions), nasal (vapors, sprays), dermal (creams, patches), or injections. (2) Groups that tested different treatment rationales or intervention components alongside with a placebo administration (i.e., Refs.^12,18,47–49^) or different amounts of placebos per day (i.e., Ref.^50^) were merged. However, study groups that tested the effect of OLPs without applying any expectation induction (here referred to as OLP −) were entered into the analyses separately. (3) If there were different comparator groups that fell within one category (e.g., several DP groups), we merged them into one node (i.e., Refs.^17,19,47,49^). (4) To assess the differences in expectation induction (e.g., through verbal suggestion or conditioning), these nodes were defined separately (e.g., OLP vs. cOLP). (5) In all the cases where participants could receive the intervention upon the conclusion of the study, we used the node WL control group. When data from the study groups were merged, we used different formulas^34^.

### Risk of bias

We assessed the risk of bias in the included studies using the Cochrane risk of bias tool 2^51^. Each study was assessed by two reviewers, and conflicts were resolved by consensus. To account for the special nature of the studies included in this NMA (i.e., that all of them were not blind), we employed some special rules: (1) If we received a rating of a “high” risk of bias in domain 4 only due to signaling question 4.5 (“Is it likely that the assessment of the outcome was influenced by knowledge of the intervention received?”), we overrode the suggestion of the algorithm for this domain and labeled it with “some concerns.” The rationale for this decision was based on the fact that a single “high” judgment in one of the four domains led to an overall high risk of bias. As a consequence, all of our included studies would have received a high overall risk rating, and we would have lost all the variance in our assessments. (2) When answering signaling questions 2.1 (“Were participants aware of their assigned intervention during the trial?”) and 4.3 (“Were the outcome assessors aware of the intervention received by the study participants?”) for the comparison OLP (i.e., aware of receiving the intervention) and DP group (i.e., not aware), we judged as if both groups were unblinded as suggested by the authors of the risk of bias tool 2^52^. (3) Because the risk of bias tool 2 requires an assessment of the level of study group comparisons within a study, multiple assessments were performed per study. However, all the assessments of each study were identical and are thus reported in a single column (see eTable 1 in the supplement).

### Statistical analysis

In order to answer our RQs we proceeded as follows: (RQ1) The effect of treatment expectations was assessed using head-to-head comparisons between OLP − and each other OLP administration-route group. (RQ2) To assess the effects of the different comparator groups (i.e., NT, TAU, WL), we compared all the OLP administration routes that were significantly better than NT with the other comparator groups. (RQ3) The OLP treatment administration routes were compared directly using head-to-head comparisons, excluding OLP −. (RQ4) Two different networks were formed, one for the clinical and one for the nonclinical population. These networks were then qualitatively compared.

Effect sizes of the interventions were calculated using standardized mean differences (*SMDs*), and their magnitudes were interpreted as small, moderate, or large, with 0.20, 0.50, and 0.80^53^. We decided to employ random-effects models rather than fixed-effects models because the included studies were expected to be heterogeneous. Network meta-analytic methods were applied within a frequentist framework using the package netmeta in R^54,55^. Results are presented as *SMD*s with corresponding 95% confidence intervals.

NMA relies on the assumption of transitivity to estimate indirect treatment effects. This assumption implies that any study participant that meets all the inclusion criteria in each network is likely, in principle, to be randomized to any of the interventions in the corresponding network. We addressed the assumption of transitivity^56^, first, by conducting two separate networks (i.e., nonclinical and clinical) to balance the distribution of potential modifiers (e.g. population) better across the comparisons and, second, by checking whether the direct and indirect treatment effects were in statistical agreement (via an assessment for inconsistency). We conducted a statistical evaluation of consistency—that is, of the agreement between direct and indirect evidence—using local (separating direct from indirect evidence^57^) as well as global (design-by-treatment interaction test^58^) approaches.

The various effects of the groups were ranked using *P* scores. *P* scores are values between 0 and 1, have an interpretation analogous to the surface under the cumulative ranking curve values^59^, and measure the extent of certainty that a treatment is better than another treatment, averaged over all competing treatments. The *P* scores resulted in a ranking of all the treatments that essentially follows the ranking of the point estimates but takes precision into account^59^.

For all the treatment comparisons in an NMA, we assumed a common between-study heterogeneity. Different statistics were used to quantify heterogeneity: the (within-design) Q statistic^54^, the between-study variance τ2, and the heterogeneity statistic *I*^*2*^^59^. The *I*^*2*^ values can be interpreted as follows: 0 to 40% might not be important; 30 to 60% may represent moderate heterogeneity; 50 to 90% substantial heterogeneity; 75 to 100% considerable heterogeneity^60^.

The certainty of the evidence for the network estimates of the efficacy outcomes was evaluated using the Grading of Recommendations Assessment, Development, and Evaluation (GRADE) ratings^56^, which were conducted in CINeMA (Confidence in Network Meta-Analysis^59^). GRADE defines the quality of a body of evidence as the study limitations, imprecision, inconsistency, indirectness, and reporting bias^56^. To assess across-study bias (reporting bias), a comparison-adjusted funnel plot and an Egger test for funnel-plot asymmetry were computed^57^. In case of asymmetry, the trim-and-fill method was used to adjust for small-study effects with NT as the reference^61,62^. Due to too few comparisons, we were not able to use the tool for assessing the risk of bias due to missing evidence in a synthesis (ROB-MEN^63^) as initially planned.

To conduct sensitivity analyses, we excluded the studies in which the risk of bias was high. We decided to choose this criterion, as all the studies had at least a moderate risk due to the fact that there was no blinding and that most outcomes were patient-reported. We also conducted sensitivity analyses to investigate if the results differed in the clinical network when the subclinical studies were excluded. Furthermore, owing to the great variance of the included conditions within each of the two networks and due to considerable heterogeneity in the nonclinical network, we performed a subgroup analysis for two broad areas: pain and psychological conditions.

## Results

A total of 12,991 records were retrieved by searching bibliographic databases and registries. After removing duplicates, 6,811 entries remained, and their titles and abstracts were screened together with an additional 21 identified records. Subsequently, 731 full texts were screened. Thirty-seven RCTs (comprising 3,021 participants) conducted between 2010 and 2022 comparing 12 interventions and three control groups met all of the eligibility criteria and were included in our analyses. A flowchart detailing the process of study identification and selection is shown in eFig. 1 in the supplement. All the studies were reported in English and included an adult sample with a mean (*SD*) age of 36 (15.3) years (range: 19–70 years). All the selected outcomes were of a continuous nature (see eTable 1 in the supplement for details on the selected outcomes). The individual characteristics of the 37 studies included in the analysis are given in eTable 1 in the supplement. All the data used in the analysis for individual studies (e.g., means and *SD*s) are available in eTable 2 in the supplement.

### Nonclinical sample

Twelve studies yielded sufficient data to be included in the analysis of the nonclinical sample (comprising 1015 participants). The sample sizes of the individual studies ranged from 21 to 151. The mean (*SD*) age of this sample was 23.6 (2.1) years (range: 20–28 years; if reported), and 67.7% of the sample population were female. Four studies examined experimentally induced pain, three itch, two sadness, one acute stress, one nausea, and one tested muscle strength. All the studies except for one were single-center studies^47^. Eight trials recruited participants from Europe (Germany, Netherlands, Switzerland, and the UK), three from North America (USA), and one from Australia. The studies had different routes of placebo administration such as nasal (4 studies), dermal (6 studies), and oral (2 studies). Ten studies used an NT control, nine included a DP, and two an OLP − condition. One study used conditioning in order to evoke positive treatment expectations; all the others used verbal suggestions.

**Figure 1:**
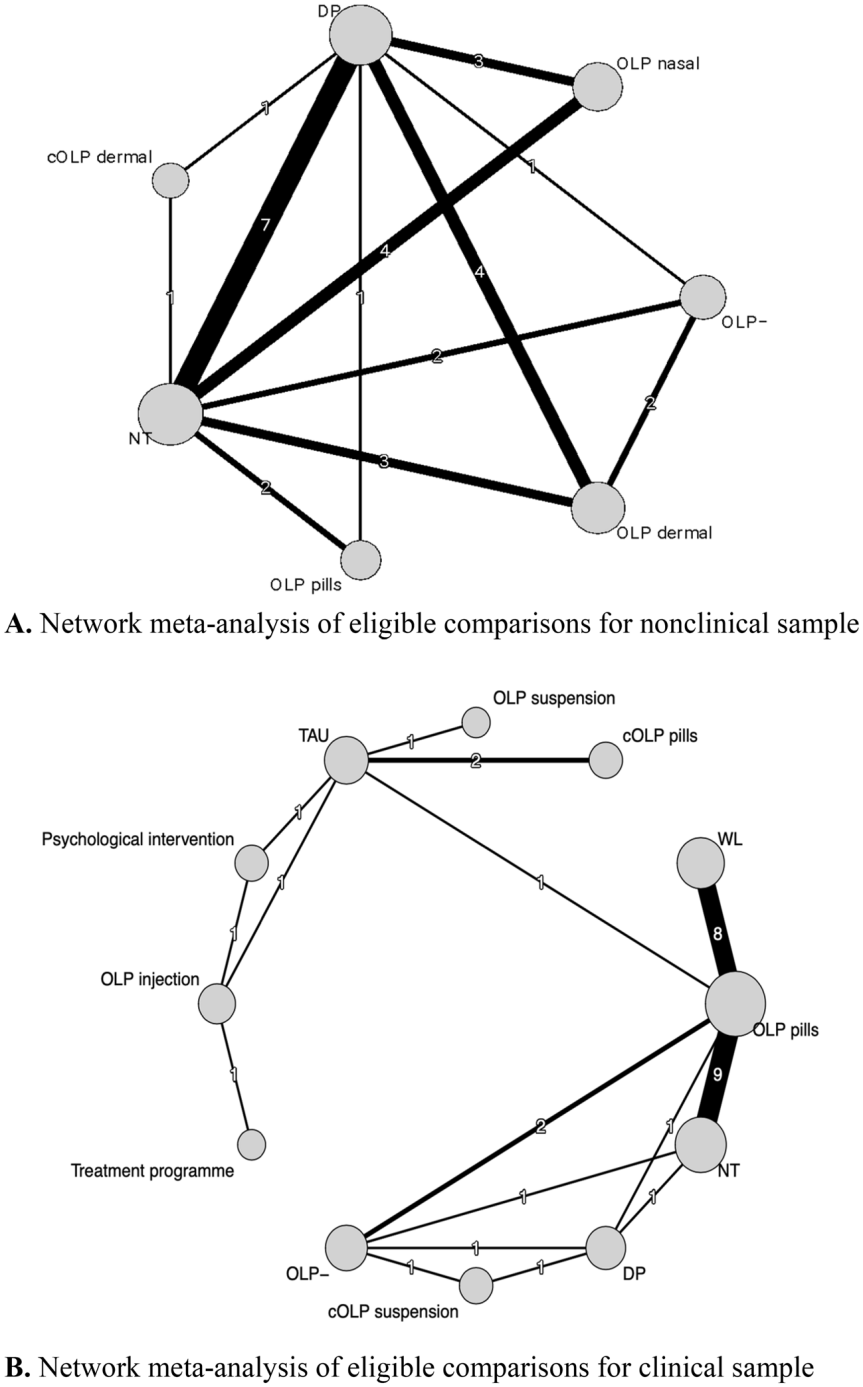
Network meta-analysis of eligible comparisons. (**A**) nonclinical. (**B**) clinical. Width of the lines is proportional to the number of trials comparing each pair of treatments/groups. *cOLP* conditioned open-label placebo, *DP* deceptive placebo, *NT* no treatment, *OLP* open-label placebo with rationale, *OLP − *open-label placebo without expectation induction, *TAU* treatment as usual, *WL* wait list.

**Figure 2:**
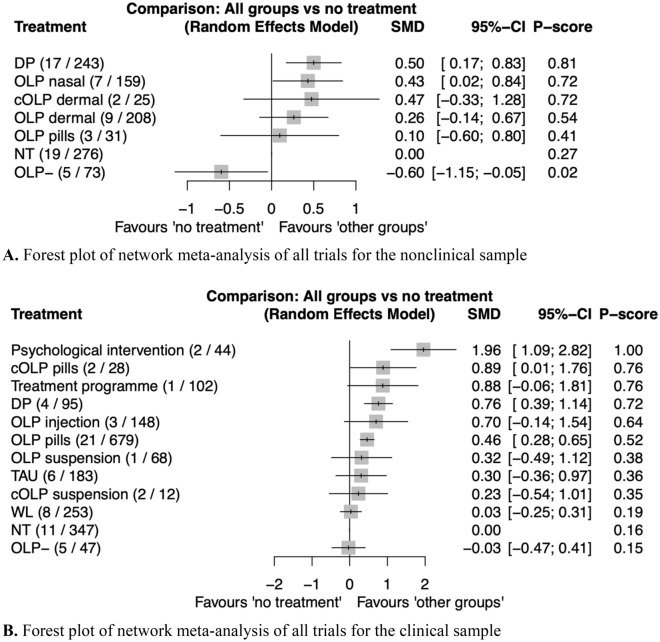
Forest plot of network meta-analysis of all trials. (**A**) Nonclinical. (**B**) Clinical. All groups were compared with no treatment (NT) as reference. The brackets behind the group names indicate (i) the number of direct comparisons with this group, (ii) the number of patients in which the intervention/control was examined. *SMD* indicates standardized mean difference. *cOLP* conditioned open-label placebo, *DP* deceptive placebo, *NT* no treatment, *OLP* open-label placebo with rationale, *OLP − *open-label placebo without expectation induction, *TAU* treatment as usual, *WL* wait list.

Figure 1A shows the network of eligible comparisons and Fig. 2A shows the forest plot of the NMA, including all the treatments and control groups, using NT as a reference. In this network, only nasal OLPs were significantly better than NT (*SMD* = 0.43, [0.02–0.84]). Dermally applied conditioned and unconditioned OLPs as well as OLP pills were not significantly better compared to NT (*SMD*s ranging from 0.10, [− 0.60–0.80], to 0.47, [− 0.33–1.28]). OLP − was worse than NT (*SMD* = − 0.60, [− 1.15 to − 0.05]). (RQ1) OLPs without an induction of treatment expectation were statistically worse compared to all the other OLP administration routes (*SMD*s ranging from − 0.86, [− 1.41 to − 0.31], to − 1.07, [− 2.02 to − 0.12]) except for the comparison to OLP pills (*SMD* = − 0.69, [− 1.57–0.19]). (RQ2) In this network, there was only one comparator (i.e., NT), so differential effects depending on which comparison group was used could not be investigated. (RQ3) The investigation of head-to-head comparisons (see eTable 3 in the supplement) of different OLP administration routes revealed no significant differences; *SMD*s ranging from 0.04, [− 0.83–0.92], to 0.38, [− 0.68–1.43].

### Clinical sample

The analysis of the clinical sample included 25 studies with 2,006 participants and sample sizes of individual studies ranging from 19 to 211. The mean (*SD*) age of this sample was 43.7 (14.9) years (range: 19–70 years), and 70.7% were female. The different populations used in the 25 included studies were the following: chronic low back pain (four studies), allergic rhinitis (three studies), cancer-related fatigue (three studies), irritable-bowel syndrome (two studies), knee osteoarthritis (two studies), major depressive disorder (two studies), acute pain (following spine surgery; one study), acute pain (spinal cord injury and polytrauma; one study), chronic low back pain + experimental pain (one study), menopausal hot flushes (one study), primary insomnia (one study), relaxation test (one study), test anxiety (one study), well-being (one study), and well-being + cognitive enhancement (one study). The mean duration of the treatment phase was 3 weeks (range: 1 day to 12 weeks). No study was multicentered. Thirteen trials recruited patients from Europe (Germany, Austria, Denmark, Portugal), eight from North America (USA), three from Asia (Japan and Israel), and one from Australia. Routes of placebo administration included injections (two studies) or oral applications, such as pills (21 studies) and suspensions (two studies). Nine studies used an NT control condition, five TAU, and eight a WL. Furthermore, two studies included a DP as a comparator group, two an OLP − group, one a psychological intervention, and one a treatment program (exercise and education intervention). Overall, three studies used a conditioning paradigm to induce positive treatment expectations.

Figure 1B depicts the clinical network with eligible comparisons, and Fig. 2B shows the forest plot of the NMA, including all the treatments and control groups, using NT as a reference. In the clinical network, conditioned and unconditioned OLP pills outperformed NT (0.89, [0.01–1.76], to 0.46, [0.28–0.65], respectively). Injected OLPs and conditioned and unconditioned OLP suspensions were not statistically better than NT (*SMD*s ranging from 0.23, [− 0.54–1.01], to 0.70, [− 0.14–1.54]). (RQ1) OLPs without the induction of treatment expectation were not statistically different from any other OLP administration route (*SMD*s ranging from − 0.26, [− 1.03–0.51], to − 0.92, [− 1.87–0.04]), except for the comparison to OLP pills; in that case, OLP − was significantly worse (*SMD* = − 0.49, [− 0.92 to − 0.07]). (RQ2) The investigation of the effects of treatment comparators showed that in addition to being better than NT, OLP pills were also significantly better than WL (*SMD* = 0.43, [0.22–0.64]), but they were not superior to TAU (*SMD* = 0.16, [− 0.48–0.80]). Also, cOLP pills were significantly better than TAU (*SMD* = 0.58, [0.02–1.15]), and marginally not significant compared with WL, yet the effect was high (*SMD* = 0.86, [− 0.02–1.74]). (RQ3) The investigation of head-to-head comparisons (see eTable 3 in the supplement) of different OLP administration routes revealed no significant differences; *SMD*s ranging from − 0.08, [− 1.18–1.01], to 0.65, [− 0.50–1.81].

The results from sensitivity analyses, adverse events, and the certainty of the evidence assessment can be found in eAppendix 6 in the supplement.

## Discussion

This systematic review and NMA of RCTs with 3,021 individuals assessed the efficacy of various OLP interventions in comparison to different types of control groups in both a nonclinical and a clinical sample. The aim was to examine whether the size of the OLP effect is different across (RQ1) treatment expectations, (RQ2) comparator groups, (RQ3) treatment administration routes, and (RQ4) nonclinical versus clinical populations. Across both population networks, a wide range of conditions were studied, pain and diverse psychological conditions being the most frequent.

OLPs without the induction of treatment expectation were statistically worse compared to the majority of OLP administration routes in the nonclinical network (*SMD* ranging from − 0.86 to − 1.07) and compared to OLP pills in the clinical network (*SMD* = − 0.49; RQ1). Also, the comparison of treatment comparator groups in the clinical network showed that OLP pills were better than WL (*SMD* = 0.43) but not better than TAU (*SMD* = 0.16; RQ2). The NMA of the nonclinical sample further revealed a significant effect of OLP administered as a spray or vapor (i.e., OLP nasal) in comparison to NT (*SMD* = 0.43). All other OLP interventions showed small to medium but insignificant *SMD*s in comparison to NT. Similar results were found for the clinical sample, where only OLP pills outperformed NT (*SMD* conditioned = 0.89; unconditioned = 0.46), and again all other administration routes showed insignificant but small to medium effects. Even though only some OLP administration routes were significantly better than NT, the direct head-to-head comparison of the different employed OLP administration routes in both networks showed no significant differences (RQ3). In contrast, the qualitative comparison of effect sizes across populations revealed different magnitudes of effects, with larger effect sizes in the clinical network (RQ4).

In the following, the observed effects will be discussed with regard to the four distinct research questions. Regarding our research question on the impact of expectation (RQ1), evidence from both networks suggests that OLP interventions delivered without the evocation of at least minimal treatment expectations were less efficacious when compared to OLP interventions with the induction of treatment expectation. This finding was especially pronounced in the nonclinical network, where OLP − was less efficacious in comparison to all other groups in the network, even to NT (*SMD* = -0.60). However, the efficacy of OLP − in the nonclinical network was solely evaluated by two trials investigating dermal placebo applications^8,18^. Nevertheless, it appears that expectancy building is an important component of OLP interventions and that simply prescribing an inert treatment is not sufficient. Hence, OLP treatments might be cost-efficient, but they are not as time-efficient as over-the-counter medicine. Possible explanations for this observation could be that participants do not feel taken seriously when they are simply told that they are receiving a placebo treatment, or that they are disappointed because they may not know about the power of placebo effects. The effects in clinical settings (where no differences between OLP − and NT were observed) might potentially be buffered by at least performing a ritual of, for example, taking pills over a period of time. In a single administration, which was often the case in the nonclinical studies, no such ritual could be established. The rationale therefore seems to be an essential and potentially indispensable component for the efficacy of OLP^64^. In this sense, OLP conditions that do not include any expectation building component could at best serve as control groups, controlling for the component of the pill. And the pill itself might not be necessary to produce positive treatment effects in OLP studies. This finding is supported by a recently published RCT on OLPs and imaginary pills^65^.

With respect to the potential impact of different comparators (RQ2), our systematic search showed that due to the experimental setting, all nonclinical studies used a NT control group. Differences across the control groups could thus only be investigated in the clinical sample. There, we identified three different comparison groups, namely NT, WL, and TAU. Comparing the effect sizes across different comparator groups showed that OLP pills were in addition to NT also significantly better than WL (*SMD* = 0.43) but not TAU (*SMD* = 0.16). In other words, this finding could imply that OLP pills are better than “nothing” but not better than “something.” The efficacy of both of these interventions seems to depend on the kind of control group used, a finding in line with psychotherapy research^20^. However, whereas WL was notably inferior to NT, in the present study both comparison groups yielded comparable effects. Conditioned OLP pills, on the other hand, were significantly better than NT as well as TAU (*SMD* = 0.58) and tended to be better than WL (*SMD* = 0.86; *not significant*). However, these findings are based on two studies only, indicating that the obtained conclusions are not entirely conclusive and should be further explored. Nevertheless, these findings suggest that comparator groups in OLP studies should be chosen carefully as the effects might differ according to the chosen comparator.

In terms of OLP administration routes (RQ3), none of the direct comparisons were statistically significant, indicating that there might not have been a difference in the effect across OLP administration routes in either sample. This finding stands in contrast to the results in DPs, where it is known that more invasive routes of administration can yield larger effects compared to less invasive procedures^27,29^. This discrepancy suggests that findings from DP research might not be valid for the field of OLP. However, *SMD*s varying up to 0.50 across administration routes suggest (especially in the clinical sample) that the current analyses might be underpowered in order to observe statistically significant differences. However, there is also reason to assume that potential differences in OLP administration routes may be obscured given that the present analyses investigated the efficacy of OLP treatments across a variety of different somatic and psychological conditions. Supporting this line of reasoning, Peerdeman et al.^66^ found that expectations toward the efficacy of different routes of administration differed for pain and itch. For example, injected medications were expected to be most effective for relieving pain and topical medications for alleviating itching. These results might reflect the impact of knowledge and prior experience on treatment expectations. Regardless, placebo experts advise against prescribing more invasive treatments to yield stronger effects, as this entails practical and ethical repercussions^1^. Especially the still small database for OLPs calls for a cautious consideration regarding the use of more invasive procedures.

In order to investigate differential effect sizes across the nonclinical and clinical samples (RQ4), we compared the findings of both networks qualitatively. We found that the effect sizes for the comparison of OLP pills to NT yielded smaller and nonsignificant effects in the nonclinical sample (i.e., *SMD* nonclinical = 0.10; clinical = 0.46). This trend was also exemplified by comparing DP to NT (*SMD* nonclinical = 0.50; clinical = 0.76). Similar observations have previously been reported for both somatic and psychological conditions. For example, studies investigating placebo analgesia have found an average effect size of 1.24 in healthy individuals and an effect size of 1.49 in patients^30^. This finding not only suggests that DPs employed in OLP studies tend to yield smaller effects as compared to studies investigating DPs only but also sheds light on the difference between the effect sizes of placebo effects across nonclinical and clinical samples. In this regard, our two networks may support the notion that effects tend to be of greater magnitude in clinical as opposed to nonclinical populations. This trend was supported by our sensitivity analysis, where the effect sizes were slightly larger when excluding subclinical studies. A potential explanation could be the more pronounced desire for relief in patients as opposed to healthy individuals^67^. In summary, this finding suggests that OLP effect sizes in clinical and subclinical contexts are larger than those in nonclinical individuals and that experimental studies on healthy individuals may underestimate the magnitude of the OLP effect in patients. However, this comparison is only qualitative in nature, so it should be further explored as part of single studies using the same study design and setting for both patients and healthy individuals.

Overall, the present analyses confirm the results of previous meta-analyses investigating the efficacy of OLPs in clinical populations, which found moderate to high effect sizes^4,5^. In contrast, the results of the nonclinical sample contradict in part the findings of Spille et al.^6^, who found a medium-sized effect for subjective outcomes for OLPs in comparison to NT. For example, when comparing OLP pills to NT, there was a small non-significant effect in our analyses. However, the difference in results could be due to the splitting into different administration routes, as nasally administered OLPs showed a medium effect (*SMD* = 0.43) compared to NT, which is consistent with the results from Spille. Further differences may also be explained by different inclusion criteria and thus a different body of studies that contributed to the results (e.g., the inclusion of subclinical studies and balanced-placebo-designs in their analysis). Remarkably, the effect sizes for OLP pills in the clinical sample in the present study were smaller in comparison to previous investigations that have only compared OLP pills to different control conditions (*SMD* = 0.46 vs. 0.88^4^ and 0.72^5^). This trend toward smaller effects across the timespan suggests that in an early stage of the research, “positive” studies were more likely to be published (a reporting bias that was also present in the clinical sample of this NMA), and that with time insignificant results were more likely to be published (time-lag bias).

This study has several strengths. First, the direct comparison of different OLP administration routes and comparators is of great importance to informing the young research field on OLPs about comparative efficacy for improving the design of future studies. The network meta-analytic approach uniquely allows investigation of the effects of different administration routes and comparators. Second, this methodology enables combining direct and indirect evidence to attain the most precise estimate of intervention differences. Third, we were able to include 14 more studies than the most recent meta-analysis on OLP in clinical conditions^5^, which strengthens the body of evidence. Fifth, the clear definition of OLPs is a strength of this analysis, as are the several sensitivity analyses that were conducted, which showed comparable results that further supported the trends of the overall analyses. Finally, the application of the same inclusion criteria for the nonclinical and the clinical sample made it possible to compare the effect sizes across both populations more reliably.

Despite its novelty and methodological advances, this study has several limitations that should be taken into account when interpreting the results. First, although the network meta-analytic approach made it possible to include 12 studies in the nonclinical network and 25 in the clinical, which represents a broad range of studies in comparison to previous analyses, the relatively small number of studies in each node and the resulting small power might have led to a lack of significance (large confidence intervals). Second, a major limitation of our NMAs is associated with the fact that most interventions were tested on fewer than 100 participants. It is therefore possible that the effect of some of these interventions was due to a so-called small-study effect: smaller trials show different, often larger, treatment effects than larger ones^68,69^. Third, substantial heterogeneity was found in our NMAs. The variety of the studied conditions, the format of the interventions (e.g., duration), and the reported outcomes differed widely, which may have contributed to the statistical heterogeneity and certainly to the clinical heterogeneity. We tried to reduce the heterogeneity by applying a very precise and strict definition of OLPs, by conducting two separate NMAs, and by choosing the most frequent outcome when several outcomes were present. Furthermore, sensitivity analyses suggest that the results remain unchanged when looking at more homogeneous subgroups, such as pain, within the networks. Fourth, although NMAs have the advantage of making use of all the available data, the indirect evidence does not directly stem from randomized comparisons^70^. Fifth, according to the GRADE framework, the within-study bias of many comparisons was assessed as “some concerns,” which can be attributed in part to methodological difficulties that arise from the nature of OLPs (i.e., participants’ being unblinded) and the fact that most outcomes were self-reported. Such self-reported outcomes can be biased (e.g., if one wants to please the examiner), which can lead to increased effects. Sixth, funnel plots and accompanying Egger’s tests indicated a risk of reporting bias for the clinical network because of the lack of small studies comparing NT to OLP pills with negative effects. Seventh, we excluded crossover studies due to analytical concerns regarding comparability with parallel trials^34^, which reduced the body of evidence to parallel trials with an accompanying loss in power. Eighth, the clinical sample also included undiagnosed subclinical conditions, which limits the comparability to other meta-analyses that only included studies with diagnosed samples (e.g., Ref.^5^). However, this was accounted for by conducting a sensitivity analysis that yielded comparable results. Tenth, the relatively early stage of OLP research made it impossible to investigate the efficacy of OLPs within distinct conditions. The present NMA therefore examined the interventions on a metalevel by lumping studies with different conditions. However, combining the effect sizes across different conditions is problematic, because it may hide placebo-responsive and placebo-nonresponsive domains. Furthermore, this might violate the requirement that NMAs only include populations that are in theory jointly randomizable. Finally, the results on the comparable efficacy of OLP administration routes might be explained by population-specific choices of treatment administration routes, obscuring potential differences within each domain.

With this NMA, we were able to identify several research gaps. First, larger studies should be conducted, as the current sample sizes are often relatively small (range: 19–211). Second, the population should be more representative. The majority of the study populations were female (70%), and especially in our nonclinical sample, the study populations were very young (mean age: 23.6 years). This complicates, among other things, the transfer of nonclinical findings to clinical populations, which was on average older (mean age: 43.7 years). Third, adverse events should be reported in a more structured and consistent manner. Because of unreported or inconsistently reported adverse events, we were not able to analyze them in the present study. Fourth, it is crucial that future studies be conducted by more independent research teams with less allegiance to OLP research. Fifth, in future studies, the control group used should be chosen deliberately, because, as our study shows, different types of control groups result in different effect sizes. Also, in future meta-analyses, the control groups should not be lumped together, as this can obscure possible treatment effects. Sixth, it would be informative for further (network) meta-analyses to distinguish between active and nonactive OLPs, which was not considered in these analyses. Seventh, further experimental studies should be designed more according to the needs of clinical populations. For instance, the OLP administration route OLP nasal (e.g., sprays) and OLP dermal (e.g., creams) have only been studied in nonclinical populations and not in clinical, possibly indicating that this route of administration is not suitable for clinical conditions. Eighth, future (network) meta-analyses should take into account that potential differences between OLP administration routes may be masked, as their effects may differ depending on the type of disease. Finally, in order to reduce within-study bias, future research should include objective outcomes and behavioral markers.

To conclude, OLPs can be beneficial compared to control conditions in nonclinical and clinical conditions. However, the magnitude of effects in our network meta-analyses was smaller compared to previous meta-analyses and our NMAs showed that effects sizes depended on several aspects. (1) The induction of positive treatment expectations appeared to be of great importance for the efficacy of OLPs. Simply prescribing an OLP seems not to be enough and might even hold the risk of being worse than receiving no treatment. (2) We found that OLP effects can vary depending on the comparator used. In other words, some interventions facilitate relief when compared to “nothing,” but their effect appears to vanish when compared to other treatments. (3) There were no differences in the effects across OLP administration routes in either sample. This finding calls for caution regarding the use of more invasive OLP procedures. (4) Finally, we identified a trend of greater effect sizes in the clinical network. Research on nonclinical samples may underestimate the magnitude of OLP effects in patients. With this NMA, we hope to contribute to the emerging research field on OLPs and to inform future studies that aim to explore ethical ways to use placebo effects for the good of patients.

## Supplementary Information

Supplementary Information.

## Data Availability

All data generated or analyzed during this study are included in this published article and the files in the Supplementary Information.
