# Supplementary material for: The roles of expectation, comparator, administration route, and population in open-label placebo effects: a network meta-analysis
Source: Sci Rep. 2023 Jul 22;13:11827. doi: 10.1038/s41598-023-39123-4 (PMC10363169; doi:10.1038/s41598-023-39123-4)
Supplement: Supplementary file 1 — Supplementary Information. [file 41598_2023_39123_MOESM1_ESM.pdf]

Supplementary Online Content

The roles of expectation, comparator, administration route, and population in open-label placebo effects: A network meta-analysis

Sarah Buergler*, Dilan Sezer*, Jens Gaab & Cosima Locher
*shared first authorship

eAppendix 1. Search strategies and hits

eAppendix 2. Hits update

eAppendix 3. GRADE ratings for each network

eAppendix 4. Details on inconsistency

eAppendix 5. PRISMA checklist

eAppendix 6. Additional Results

eFigure 1. Flow chart

eFigure 2. Funnel plots with accompanying Egger test

eFigure 3. Plots of low and moderate risk of bias only (sensitivity analysis)

eFigure 4. Plots of clinical network without subclinical trials (sensitivity analysis)

eFigure 5. Plots of pain trials (sensitivity analysis)

eFigure 6. Plots of psychological trials (sensitivity analysis)

eTable 1. Demographics and study characteristics

eTable 2. Individual study data

eTable 3. Head to head comparisons

eReferences

eAppendix 1. Search strategies and hits

Medline Ovid

(20210202; 956 hits)

(((placebo* or sham) adj2 (open-label* or told or nondecept* or non-decept* or nonconceal* or non conceal* or unconceal* or unblind* or nonblind* or non blind* or without decept* or without conceal* or without blind*)).ti,ab,kw,kf. or open placebo*.ti,ab,kw,kf. or ((placebos/ or Placebo Effect/) and (open-label* or told or nondecept* or non-decept* or nonconceal* or non conceal* or unconceal* or unblind* or nonblind* or non blind* or without decept* or without conceal* or without blind*).ti,ab.)) and (exp Random Allocation/ or exp Randomized Controlled Trial/ or exp Randomized Controlled Trials as Topic/ or RCT or (randomiz* or randomis*).ti,ab. or ((controlled clinical or non-inferiority or noninferiority or superiority or equivalence or pragmatic) ADJ2 trial$).ti,ab.)

Embase Ovid

(20210202; 5,487 hits)

(((placebo* or sham) adj2 (open-label* or told or nondecept* or non-decept* or nonconceal* or non conceal* or unconceal* or unblind* or nonblind* or non blind* or without decept* or without conceal* or without blind*)).ti,ab,kw. or open placebo*.ti,ab,kw. or ((placebo/ or Placebo Effect/ or sham procedure/) and (Open study/ or (open-label* or told or nondecept* or non-decept* or nonconceal* or non conceal* or unconceal* or unblind* or nonblind* or non blind* or without decept* or without conceal* or without blind*).ti,ab.))) and (randomization/ or exp randomized controlled trial/ or randomized controlled trial topic/ or "randomized controlled trial (topic)"/ or RCT or (randomiz* or randomis*).ti,ab. or ((controlled clinical or non-inferiority or noninferiority or superiority or equivalence or pragmatic) ADJ2 trial$).ti,ab.)

NOT (conference abstract or conference review).pt

CINAHL Ebsco

(20210202; 589 hits)

((((TI placebo* OR AB placebo*) OR (TI sham OR AB sham)) N2 ((TI open-label* OR AB open-label*) OR (TI told OR AB told) OR (TI nondecept* OR AB nondecept*) OR (TI non-decept* OR AB non-decept*) OR (TI nonconceal* OR AB nonconceal*) OR (TI "non conceal*" OR AB "non conceal*") OR (TI unconceal* OR AB unconceal*) OR (TI unblind* OR AB unblind*) OR (TI nonblind* OR AB nonblind*) OR (TI "non blind*" OR AB "non blind*") OR (TI "without decept*" OR AB "without decept*") OR (TI "without conceal*" OR AB "without conceal*") OR (TI "without blind*" OR AB "without blind*"))) OR (TI "open placebo*" OR AB "open placebo*") OR (((MH "placebos") OR (MH "Placebo Effect")) AND ((TI open-label* OR AB open-label*) OR (TI told OR AB told) OR (TI nondecept* OR AB nondecept*) OR (TI non-decept* OR AB non-decept*) OR (TI nonconceal* OR AB nonconceal*) OR (TI "non conceal*" OR AB "non conceal*") OR (TI unconceal* OR AB unconceal*) OR (TI unblind* OR AB unblind*) OR (TI nonblind* OR AB nonblind*) OR (TI "non blind*" OR AB "non blind*") OR (TI "without decept*" OR AB "without decept*") OR (TI "without conceal*" OR AB "without conceal*") OR (TI "without blind*" OR AB "without blind*"))))

PsycINFO Ovid

(20210202; 406 hits)

(((placebo* or sham) adj2 (open-label* or told or nondecept* or non-decept* or nonconceal* or non conceal* or unconceal* or unblind* or nonblind* or non blind* or without decept* or without conceal* or without blind*)).ti,ab. or open placebo*.ti,ab. or (placebo/ and (open-label* or told or nondecept* or non-decept* or

nonconceal* or non conceal* or unconceal* or unblind* or nonblind* or non blind* or without decept* or without conceal* or without blind*).ti,ab.))

eAppendix 2. Hits update

Medline Ovid

(20210201 bis 20220608; 66 hits)

limit *SEARCH* to dt=20210201-20220608

Embase Ovid

(20210201 bis 20220608; 640 hits)

limit *SEARCH* to dc=20210201-20220608

CINAHL Ebsco

(20210201 bis 20220608; 38 hits)

PsycINFO Ovid

(20210201 bis 20220608; 43 hits)

limit *SEARCH* to up=20210201-20220608

eAppendix 3. GRADE Ratings for each network

We used the Grading of Recommendations Assessment, Development, and Evaluation ratings (GRADE[1]) and the corresponding web application to apply this framework[2,3]. The certainty of evidence for each network estimate was assessed according to the following criteria:

Study limitations (Within study bias): The overall risk of bias of each study was categorized. According to the Cochrane Risk of Bias tool 2[4], we rated five risk of bias domains. We then used the contribution matrix to calculate the percentage of contribution from each study, and finally assessed the study limitation for each network estimate based on the weighted average risk of bias of the contributing studies. We selected the rule “Average Risk of Bias” in order to calculate the within study bias.

Reporting bias (Across studies bias): Since each of our comparisons had less than 10 comparisons, we could not use the ROB-MEN[5] tool to assess reporting bias. Therefore, a comparison-adjusted funnel plot with accompanying Egger test for asymmetry was conducted and used as a basis for the judgment.

Indirectness: We judged that there was no concern in this domain as the included studies matched our inclusion criteria and study questions.

Imprecision: In line with previous analyses[6], we considered a clinically meaningful threshold for standardized mean difference (SMD) to be 0.20.

Heterogeneity: We evaluated the degree of concerns through comparing the clinical inference based on the 95% confidence intervals (CI), the latter reflecting the degree of heterogeneity. Appling the same clinical inference framework as for imprecision, we saw no concerns in heterogeneity when the two judgements matched (e.g. no concern based on 95% CI and no concern based on 95% PI), some concerns when they differed by one degree (e.g. no concern based on 95% CI but some concerns based on 95% PI), and major concerns when they differed by two degrees (e.g. no concern based on 95% CI but major concerns based on 95% PI).

Incoherence (Inconsistency): For inconsistency, we looked at the results of side splitting and we saw major concerns when $p<0.05$ but no concern otherwise.

Nonclinical network

We found some concerns for *within-study bias* (i.e., study limitations) for all pairwise comparisons, due to the nature of the studies being unblind and most outcomes being self-reported. In terms of the *across-study bias* (i.e., reporting bias), the Egger test for funnel plot asymmetry was non-significant (p = .666) indicating that selection bias is not a big threat to the network meta-analysis. There was no concern for *indirectness*, since the included studies all matched our study questions. Evaluating *imprecision*, we found that all statistically significant comparisons revealed a clinically significant effect size. Furthermore, we examined *heterogeneity*, which is represented by the 95% prediction interval for each individual comparison. For all statistically significant comparisons there were at least some concerns regarding heterogeneity, indicating that there is a high variability of effects. Furthermore, we found no evidence for substantial and statistically significant heterogeneity in the network as a whole (within design Q = 2.27, p = .811, tau^2 = 0.13; I^2 = 66%). Finally, there was no evidence of incoherence between the direct and indirect evidence, i.e., all p-values were above 5%. For those comparisons where only indirect evidence was available incoherence was set to major concerns. Also, we identified evidence of inconsistency in the NMA when calculating the global design-by-treatment interaction test (between designs Q = 41.43, p < .001).

eAppendix 3A. GRADE ratings from web application for nonclinical network

Comparison	Number of Studies	Within-study bias	Reporting bias	Indirectness	Imprecision	Heterogeneity	Incoherence
Mixed evidence							
DP vs NT	7	Some concerns	Low risk	No concerns	No concerns	Major concerns	No concerns
DP vs OLP dermal	4	Some concerns	Low risk	No concerns	Some concerns	Some concerns	No concerns
DP vs OLP nasal	3	Some concerns	Low risk	No concerns	Major concerns	No concerns	No concerns
DP vs OLP pills	1	Some concerns	Low risk	No concerns	Major concerns	No concerns	No concerns
DP vs OLP-	1	Some concerns	Low risk	No concerns	No concerns	No concerns	No concerns
DP vs cOLP dermal	1	Some concerns	Low risk	No concerns	Major concerns	No concerns	No concerns
NT vs OLP dermal	3	Some concerns	Low risk	No concerns	Some concerns	Some concerns	No concerns
NT vs OLP nasal	4	Some concerns	Low risk	No concerns	No concerns	Major concerns	No concerns
NT vs OLP pills	2	Some concerns	Low risk	No concerns	Major concerns	No concerns	No concerns
NT vs OLP-	2	Some concerns	Low risk	No concerns	No concerns	Major concerns	No concerns
NT vs cOLP dermal	1	Some concerns	Low risk	No concerns	Major concerns	No concerns	No concerns
OLP dermal vs OLP-	2	Some concerns	Low risk	No concerns	No concerns	Some concerns	No concerns

		Indirect evidence					
**OLP dermal vs OLP nasal**	--	Some concerns	Low risk	No concerns	Major concerns	No concerns	Major concerns
**OLP dermal vs OLP pills**	--	Some concerns	Low risk	No concerns	Major concerns	No concerns	Major concerns
**OLP dermal vs cOLP dermal**	--	Some concerns	Low risk	No concerns	Major concerns	No concerns	Major concerns
**OLP nasal vs OLP pills**	--	Some concerns	Low risk	No concerns	Major concerns	No concerns	Major concerns
**OLP nasal vs OLP-**	--	Some concerns	Low risk	No concerns	No concerns	Some concerns	Major concerns
**OLP nasal vs cOLP dermal**	--	Some concerns	Low risk	No concerns	Major concerns	No concerns	Major concerns
**OLP pills vs OLP-**	--	Some concerns	Low risk	No concerns	Some concerns	Some concerns	Major concerns
**OLP pills vs cOLP dermal**	--	Some concerns	Low risk	No concerns	Major concerns	No concerns	Major concerns
**OLP- vs cOLP dermal**	--	Some concerns	Low risk	No concerns	No concerns	Major concerns	Major concerns

Clinical network

We found some concerns for *within-study bias* (i.e., study limitations) for most pairwise comparisons, due to the nature of the studies being unblind and most outcomes being self-reported. In terms of the *across-study bias* (i.e., reporting bias), the Egger test for funnel plot asymmetry was significant (p = .036) indicating that reporting bias is a threat to the network meta-analysis. There was no concern for *indirectness*, since the included studies all matched our study questions. Evaluating *imprecision*, we found that all statistically significant comparisons revealed a clinically significant effect size, except for two comparisons (cOLP suspension vs. DP, cOLP suspension vs. OLP-) where we found major concerns regarding the clinical significance of observed effects. Furthermore, we examine *heterogeneity*, which is represented by the 95% prediction interval for each individual comparison. For three statistically significant comparisons (TAU vs. cOLP pills, NT vs. cOLP pills, OLP- vs. OLP pills) there were some concerns regarding heterogeneity, indicating that there is some variability of effects. All other significant comparisons revealed no concerns. Furthermore, we found no evidence for substantial and statistically significant heterogeneity in the network as a whole (within design Q = 12.62, p = .557, tau^2 = 0.024; I^2 = 26.5%). Finally, there was evidence of incoherence between the direct and indirect evidence in three comparisons, i.e., cOLP suspension vs. OLP-, cOLP suspension vs. DP, DP vs. OLP-. For those comparisons where only indirect evidence was available incoherence was set to major concerns. Also, we identified evidence of inconsistency in the NMA when calculating the global design-by-treatment interaction test (between designs Q = 11.86, p = .018).

eAppendix 3B. GRADE ratings from web application for clinical network

Comparison	Number of Studies	Within-study bias	Reporting bias	Indirectness	Imprecision	Heterogeneity	Incoherence
			Mixed evidence				
DP vs NT	1	Some concerns	Some concerns	No concerns	No concerns	No concerns	Some concerns
DP vs OLP pills	1	Some concerns	Some concerns	No concerns	Some concerns	Some concerns	No concerns
DP vs OLP-	1	Some concerns	Some concerns	No concerns	No concerns	No concerns	Major concerns
DP vs cOLP suspension	1	Some concerns	Some concerns	No concerns	Major concerns	No concerns	Major concerns
NT vs OLP pills	9	Some concerns	Some concerns	No concerns	No concerns	No concerns	No concerns
NT vs OLP-	1	Some concerns	Some concerns	No concerns	Major concerns	No concerns	No concerns
OLP injection vs Psychological intervention	1	No concerns	Some concerns	No concerns	No concerns	No concerns	Major concerns
OLP injection vs TAU	1	No concerns	Some concerns	No concerns	Some concerns	Some concerns	Major concerns
OLP injection vs Treatment programme	1	Some concerns	Some concerns	No concerns	Major concerns	No concerns	Major concerns
OLP pills vs OLP-	2	Some concerns	Some concerns	No concerns	No concerns	Some concerns	Major concerns
OLP pills vs TAU	1	Some concerns	Some concerns	No concerns	Major concerns	No concerns	Major concerns
OLP pills vs WL	8	Some concerns	Some concerns	No concerns	No concerns	No concerns	Major concerns
OLP suspension vs TAU	1	Some concerns	Some concerns	No concerns	Major concerns	No concerns	Major concerns
OLP- vs cOLP suspension	1	Some concerns	Some concerns	No concerns	Major concerns	No concerns	Major concerns
Psychological intervention vs TAU	1	No concerns	Some concerns	No concerns	No concerns	No concerns	Major concerns
TAU vs cOLP pills	2	Some concerns	Some concerns	No concerns	No concerns	Some concerns	Major concerns

		Indirect evidence					
**DP vs OLP injection**	--	Some concerns	Some concerns	No concerns	Major concerns	No concerns	Major concerns
**DP vs OLP suspension**	--	Some concerns	Some concerns	No concerns	Major concerns	No concerns	Major concerns
**DP vs Psychological intervention**	--	Some concerns	Some concerns	No concerns	No concerns	No concerns	Major concerns
**DP vs TAU**	--	Some concerns	Some concerns	No concerns	Major concerns	No concerns	Major concerns
**DP vs Treatment programme**	--	Some concerns	Some concerns	No concerns	Major concerns	No concerns	Major concerns
**DP vs WL**	--	Some concerns	Some concerns	No concerns	No concerns	No concerns	Major concerns
**DP vs cOLP pills**	--	Some concerns	Some concerns	No concerns	Major concerns	No concerns	Major concerns
**NT vs OLP injection**	--	Some concerns	Some concerns	No concerns	Some concerns	Some concerns	Major concerns
**NT vs OLP suspension**	--	Some concerns	Some concerns	No concerns	Major concerns	No concerns	Major concerns
**NT vs Psychological intervention**	--	Some concerns	Some concerns	No concerns	No concerns	No concerns	Major concerns
**NT vs TAU**	--	Some concerns	Some concerns	No concerns	Major concerns	No concerns	Major concerns
**NT vs Treatment programme**	--	Some concerns	Some concerns	No concerns	Some concerns	No concerns	Major concerns
**NT vs WL**	--	Some concerns	Some concerns	No concerns	Major concerns	No concerns	Major concerns
**NT vs cOLP pills**	--	Some concerns	Some concerns	No concerns	No concerns	Some concerns	Major concerns
**NT vs cOLP suspension**	--	Some concerns	Some concerns	No concerns	Major concerns	No concerns	Major concerns
**OLP injection vs OLP pills**	--	No concerns	Some concerns	No concerns	Major concerns	No concerns	Major concerns
**OLP injection vs OLP suspension**	--	No concerns	Some concerns	No concerns	Major concerns	No concerns	Major concerns
**OLP injection vs OLP-**	--	Some concerns	Some concerns	No concerns	Some concerns	Some concerns	Major concerns
**OLP injection vs WL**	--	Some concerns	Some concerns	No concerns	Some concerns	Some concerns	Major concerns
**OLP injection vs cOLP pills**	--	Some concerns	Some concerns	No concerns	Major concerns	No concerns	Major concerns

**OLP injection vs cOLP suspension**	--	Some concerns	Some concerns	No concerns	Major concerns	No concerns	Major concerns
**OLP pills vs OLP suspension**	--	Some concerns	Some concerns	No concerns	Major concerns	No concerns	Major concerns
**OLP pills vs Psychological intervention**	--	No concerns	Some concerns	No concerns	No concerns	No concerns	Major concerns
**OLP pills vs Treatment programme**	--	Some concerns	Some concerns	No concerns	Major concerns	No concerns	Major concerns
**OLP pills vs cOLP pills**	--	Some concerns	Some concerns	No concerns	Major concerns	No concerns	Major concerns
**OLP pills vs cOLP suspension**	--	Some concerns	Some concerns	No concerns	Major concerns	No concerns	Major concerns
**OLP suspension vs OLP-**	--	Some concerns	Some concerns	No concerns	Major concerns	No concerns	Major concerns
**OLP suspension vs Psychological intervention**	--	No concerns	Some concerns	No concerns	No concerns	No concerns	Major concerns
**OLP suspension vs Treatment programme**	--	Some concerns	Some concerns	No concerns	Major concerns	No concerns	Major concerns
**OLP suspension vs WL**	--	Some concerns	Some concerns	No concerns	Major concerns	No concerns	Major concerns
**OLP suspension vs cOLP pills**	--	Some concerns	Some concerns	No concerns	Some concerns	Some concerns	Major concerns

**OLP suspension vs cOLP suspension**	--	Some concerns	Some concerns	No concerns	Major concerns	No concerns	Major concerns
**OLP- vs Psychological intervention**	--	Some concerns	Some concerns	No concerns	No concerns	No concerns	Major concerns
**OLP- vs TAU**	--	Some concerns	Some concerns	No concerns	Major concerns	No concerns	Major concerns
**OLP- vs Treatment programme**	--	Some concerns	Some concerns	No concerns	Some concerns	Some concerns	Major concerns
**OLP- vs WL**	--	Some concerns	Some concerns	No concerns	Major concerns	No concerns	Major concerns
**OLP- vs cOLP pills**	--	Some concerns	Some concerns	No concerns	Some concerns	No concerns	Major concerns
**Psychological intervention vs Treatment programme**	--	No concerns	Some concerns	No concerns	No concerns	No concerns	Major concerns
**Psychological intervention vs WL**	--	Some concerns	Some concerns	No concerns	No concerns	No concerns	Major concerns
**Psychological intervention vs cOLP pills**	--	Some concerns	Some concerns	No concerns	No concerns	No concerns	Major concerns
**Psychological intervention vs cOLP suspension**	--	Some concerns	Some concerns	No concerns	No concerns	No concerns	Major concerns
**TAU vs Treatment programme**	--	No concerns	Some concerns	No concerns	Some concerns	Some concerns	Major concerns

**TAU vs WL**	--	Some concerns	Some concerns	No concerns	Major concerns	No concerns	Major concerns
**TAU vs cOLP suspension**	--	Some concerns	Some concerns	No concerns	Major concerns	No concerns	Major concerns
**Treatment programme vs WL**	--	Some concerns	Some concerns	No concerns	Some concerns	Some concerns	Major concerns
**Treatment programme vs cOLP pills**	--	Some concerns	Some concerns	No concerns	Major concerns	No concerns	Major concerns
**Treatment programme vs cOLP suspension**	--	Some concerns	Some concerns	No concerns	Major concerns	No concerns	Major concerns
**WL vs cOLP pills**	--	Some concerns	Some concerns	No concerns	Some concerns	No concerns	Major concerns
**WL vs cOLP suspension**	--	Some concerns	Some concerns	No concerns	Major concerns	No concerns	Major concerns
**cOLP pills vs cOLP suspension**	--	Some concerns	Some concerns	No concerns	Major concerns	No concerns	Major concerns

eAppendix 4. Details on inconsistency

eAppendix 4A. Nonclinical network – local approach

```
Separate indirect from direct evidence (SIDE) using back-calculation method

Random effects model:

            comparison k prop   nma direct indir.  Diff     z p-value
        cOLP dermal:DP 1 0.78 -0.03  -0.09   0.20 -0.29 -0.29  0.7720
        cOLP dermal:NT 1 0.78  0.47   0.54   0.25  0.29  0.29  0.7720
 cOLP dermal:OLP dermal 0    0  0.21      .   0.21     .     .       .
  cOLP dermal:OLP nasal 0    0  0.04      .   0.04     .     .       .
  cOLP dermal:OLP pills 0    0  0.38      .   0.38     .     .       .
      cOLP dermal:OLP- 0    0  1.07      .   1.07     .     .       .
                 DP:NT 7 0.85  0.50   0.47   0.66 -0.19 -0.42  0.6773
         DP:OLP dermal 4 0.77  0.24   0.10   0.70 -0.61 -1.28  0.2011
          DP:OLP nasal 3 0.74  0.07   0.21  -0.33  0.55  1.09  0.2751
          DP:OLP pills 1 0.34  0.40  -0.01   0.62 -0.63 -0.79  0.4306
               DP:OLP- 1 0.43  1.10   1.44   0.84  0.60  1.02  0.3078
         OLP dermal:NT 3 0.70  0.26   0.20   0.41 -0.21 -0.47  0.6404
          OLP nasal:NT 4 0.87  0.43   0.50  -0.03  0.53  0.86  0.3890
          OLP pills:NT 2 0.91  0.10  -0.00   1.09 -1.09 -0.89  0.3747
               OLP-:NT 2 0.83 -0.60  -0.70  -0.12 -0.57 -0.77  0.4386
   OLP dermal:OLP nasal 0    0 -0.17      .  -0.17     .     .       .
   OLP dermal:OLP pills 0    0  0.17      .   0.17     .     .       .
       OLP dermal:OLP- 2 0.85  0.86   0.86   0.85  0.01  0.01  0.9882
    OLP nasal:OLP pills 0    0  0.33      .   0.33     .     .       .
        OLP nasal:OLP- 0    0  1.03      .   1.03     .     .       .
        OLP pills:OLP- 0    0  0.69      .   0.69     .     .       .

Legend:
 comparison - Treatment comparison
 k          - Number of studies providing direct evidence
 prop       - Direct evidence proportion
 nma        - Estimated treatment effect (SMD) in network meta-analysis
 direct     - Estimated treatment effect (SMD) derived from direct evidence
 indir.     - Estimated treatment effect (SMD) derived from indirect evidence
 Diff       - Difference between direct and indirect treatment estimates
 z          - z-value of test for disagreement (direct versus indirect)
 p-value    - p-value of test for disagreement (direct versus indirect)
```

eAppendix 4B. Nonclinical network – global approach

```
Q statistics to assess homogeneity / consistency

                    Q df  p-value
Total           43.69 15   0.0001
Within designs   2.27  5   0.8112
Between designs 41.43 10 < 0.0001

Design-specific decomposition of within-designs Q statistic

          Design    Q df p-value
   DP:OLP dermal 0.12  1  0.7324
 NT:DP:OLP nasal 2.15  4  0.7083

Between-designs Q statistic after detaching of single designs

       Detached design     Q df  p-value
         DP:OLP dermal 40.30  9 < 0.0001
          NT:OLP nasal 37.82  9 < 0.0001
          NT:OLP pills 41.14  9 < 0.0001
      NT:DP:OLP dermal 40.17  8 < 0.0001
 NT:DP:OLP dermal:OLP-  6.98  7   0.4306
       NT:DP:OLP nasal 35.92  8 < 0.0001
       NT:DP:OLP pills 40.19  8 < 0.0001
    NT:OLP dermal:OLP- 14.51  8   0.0693

Q statistic to assess consistency under the assumption of
a full design-by-treatment interaction random effects model

                    Q df  p-value tau.within tau2.within
Between designs 41.43 10 < 0.0001          0           0
```

eAppendix 4C. Clinical network – local approach

Separate indirect from direct evidence (SIDE) using back-calculation method

Random effects model:

comparison	k	prop	nma	direct	indir.	Diff	z	p-value
cOLP pills:cOLP suspension	0	0	0.65	.	0.65	.	.	.
cOLP pills:DP	0	0	0.12	.	0.12	.	.	.
cOLP pills:NT	0	0	0.89	.	0.89	.	.	.
cOLP pills:OLP injection	0	0	0.19	.	0.19	.	.	.
cOLP pills:OLP pills	0	0	0.42	.	0.42	.	.	.
cOLP pills:OLP suspension	0	0	0.57	.	0.57	.	.	.
cOLP pills:OLP-	0	0	0.92	.	0.92	.	.	.
cOLP pills:Psychological intervention	0	0	-1.07	.	-1.07	.	.	.
cOLP pills:TAU	2	1.00	0.58	0.58	.	.	.	.
cOLP pills:Treatment programme	0	0	0.01	.	0.01	.	.	.
cOLP pills:WL	0	0	0.86	.	0.86	.	.	.
cOLP suspension:DP	1	0.89	-0.53	-0.93	2.58	-3.51	-2.98	0.0029
cOLP suspension:NT	0	0	0.23	.	0.23	.	.	.
cOLP suspension:OLP injection	0	0	-0.47	.	-0.47	.	.	.
cOLP suspension:OLP pills	0	0	-0.23	.	-0.23	.	.	.
cOLP suspension:OLP suspension	0	0	-0.08	.	-0.08	.	.	.
cOLP suspension:OLP-	1	0.78	0.26	0.89	-1.91	2.80	2.98	0.0029
cOLP suspension:Psychological intervention	0	0	-1.72	.	-1.72	.	.	.
cOLP suspension:TAU	0	0	-0.07	.	-0.07	.	.	.
cOLP suspension:Treatment programme	0	0	-0.65	.	-0.65	.	.	.
cOLP suspension:WL	0	0	0.20	.	0.20	.	.	.
DP:NT	1	0.69	0.76	0.52	1.31	-0.79	-1.92	0.0546
DP:OLP injection	0	0	0.06	.	0.06	.	.	.
DP:OLP pills	1	0.68	0.30	0.11	0.72	-0.61	-1.51	0.1298
DP:OLP suspension	0	0	0.45	.	0.45	.	.	.
DP:OLP-	1	0.35	0.79	1.82	0.24	1.58	2.98	0.0029
DP:Psychological intervention	0	0	-1.19	.	-1.19	.	.	.
DP:TAU	0	0	0.46	.	0.46	.	.	.
DP:Treatment programme	0	0	-0.11	.	-0.11	.	.	.
DP:WL	0	0	0.73	.	0.73	.	.	.
OLP injection:NT	0	0	0.70	.	0.70	.	.	.
OLP pills:NT	9	0.99	0.46	0.47	-0.39	0.86	1.09	0.2749
OLP suspension:NT	0	0	0.32	.	0.32	.	.	.
OLP-:NT	1	0.33	-0.03	0.42	-0.26	0.68	1.42	0.1564
Psychological intervention:NT	0	0	1.96	.	1.96	.	.	.
TAU:NT	0	0	0.30	.	0.30	.	.	.
Treatment programme:NT	0	0	0.88	.	0.88	.	.	.
WL:NT	0	0	0.03	.	0.03	.	.	.
OLP injection:OLP pills	0	0	0.24	.	0.24	.	.	.
OLP injection:OLP suspension	0	0	0.38	.	0.38	.	.	.
OLP injection:OLP-	0	0	0.73	.	0.73	.	.	.
OLP injection:Psychological intervention	1	1.00	-1.26	-1.26	.	.	.	.
OLP injection:TAU	1	1.00	0.39	0.39	.	.	.	.
OLP injection:Treatment programme	1	1.00	-0.18	-0.18	.	.	.	.
OLP injection:WL	0	0	0.67	.	0.67	.	.	.
OLP pills:OLP suspension	0	0	0.15	.	0.15	.	.	.
OLP pills:OLP-	2	0.69	0.49	0.23	1.07	-0.84	-1.77	0.0770
OLP pills:Psychological intervention	0	0	-1.49	.	-1.49	.	.	.
OLP pills:TAU	1	1.00	0.16	0.16	.	.	.	.
OLP pills:Treatment programme	0	0	-0.41	.	-0.41	.	.	.
OLP pills:WL	8	1.00	0.43	0.43	.	.	.	.
OLP suspension:OLP-	0	0	0.35	.	0.35	.	.	.
OLP suspension:Psychological intervention	0	0	-1.64	.	-1.64	.	.	.
OLP suspension:TAU	1	1.00	0.01	0.01	.	.	.	.
OLP suspension:Treatment programme	0	0	-0.56	.	-0.56	.	.	.
OLP suspension:WL	0	0	0.28	.	0.28	.	.	.
OLP-:Psychological intervention	0	0	-1.99	.	-1.99	.	.	.
OLP-:TAU	0	0	-0.34	.	-0.34	.	.	.
OLP-:Treatment programme	0	0	-0.91	.	-0.91	.	.	.
OLP-:WL	0	0	-0.06	.	-0.06	.	.	.
Psychological intervention:TAU	1	1.00	1.65	1.65	.	.	.	.
Psychological intervention:Treatment programme	0	0	1.08	.	1.08	.	.	.
Psychological intervention:WL	0	0	1.92	.	1.92	.	.	.
TAU:Treatment programme	0	0	-0.57	.	-0.57	.	.	.
TAU:WL	0	0	0.27	.	0.27	.	.	.
Treatment programme:WL	0	0	0.85	.	0.85	.	.	.

Legend:

comparison - Treatment comparison
k - Number of studies providing direct evidence
prop - Direct evidence proportion
nma - Estimated treatment effect (SMD) in network meta-analysis
direct - Estimated treatment effect (SMD) derived from direct evidence
indir. - Estimated treatment effect (SMD) derived from indirect evidence
Diff - Difference between direct and indirect treatment estimates
z - z-value of test for disagreement (direct versus indirect)
p-value - p-value of test for disagreement (direct versus indirect)

eAppendix 4D. Clinical network – global approach

```
Q statistics to assess homogeneity / consistency

                    Q df p-value
Total           24.48 18  0.1400
Within designs  12.62 14  0.5569
Between designs 11.86  4  0.0184

Design-specific decomposition of within-designs Q statistic

         Design    Q df p-value
 cOLP pills:TAU 0.27  1  0.6041
   NT:OLP pills 4.40  6  0.6224
   OLP pills:WL 7.94  7  0.3375

Between-designs Q statistic after detaching of single designs

   Detached design     Q df p-value
      NT:OLP pills 11.86  3  0.0079
    OLP pills:OLP-  9.45  3  0.0239
   NT:DP:OLP pills  0.96  2  0.6199
 NT:OLP pills:OLP-  9.30  2  0.0096

Q statistic to assess consistency under the assumption of
a full design-by-treatment interaction random effects model

                    Q df p-value tau.within tau2.within
Between designs 11.86  4  0.0184          0           0
```

eAppendix 5. PRISMA checklist

Section/Topic	Item #	Checklist Item	Reported on Page #
**TITLE**			
Title	1	Identify the report as a systematic review *incorporating a network meta-analysis (or related form of meta-analysis).*	p.1
**ABSTRACT**			
Structured summary	2	Provide a structured summary including, as applicable:<br>**Background:** main objectives<br>**Methods:** data sources; study eligibility criteria, participants, and interventions; study appraisal; and *synthesis methods, such as network meta-analysis.*<br>**Results:** number of studies and participants identified; summary estimates with corresponding confidence/credible intervals; *treatment rankings may also be discussed. Authors may choose to summarize pairwise comparisons against a chosen treatment included in their analyses for brevity.*<br>**Discussion/Conclusions:** limitations; conclusions and implications of findings.<br>**Other:** primary source of funding; systematic review registration number with registry name.	p.2
**INTRODUCTION**			
Rationale	3	Describe the rationale for the review in the context of what is already known*, including mention of why a network meta-analysis has been conducted.*	p.3-4
Objectives	4	Provide an explicit statement of questions being addressed, with reference to participants, interventions, comparisons, outcomes, and study design (PICOS).	p.4
**METHODS**			
Protocol and registration	5	Indicate whether a review protocol exists and if and where it can be accessed (e.g., Web address); and, if available, provide registration information, including registration number.	p.6
Eligibility criteria	6	Specify study characteristics (e.g., PICOS, length of follow-up) and report characteristics (e.g., years considered, language, publication status) used as criteria for eligibility, giving rationale. *Clearly describe eligible treatments included in the treatment network, and note whether any have been clustered or merged into the same node (with justification).*	p.5-6

Information sources	7	Describe all information sources (e.g., databases with dates of coverage, contact with study authors to identify additional studies) in the search and date last searched.	p.5
Search	8	Present full electronic search strategy for at least one database, including any limits used, such that it could be repeated.	p.5<br>eAppendix 1
Study selection	9	State the process for selecting studies (i.e., screening, eligibility, included in systematic review, and, if applicable, included in the meta-analysis).	p.5-6
Data collection process	10	Describe method of data extraction from reports (e.g., piloted forms, independently, in duplicate) and any processes for obtaining and confirming data from investigators.	p.6-7
Data items	11	List and define all variables for which data were sought (e.g., PICOS, funding sources) and any assumptions and simplifications made.	p.5-7
**Geometry of the network**	**S1**	Describe methods used to explore the geometry of the treatment network under study and potential biases related to it. This should include how the evidence base has been graphically summarized for presentation, and what characteristics were compiled and used to describe the evidence base to readers.	p.7-9
Risk of bias within individual studies	12	Describe methods used for assessing risk of bias of individual studies (including specification of whether this was done at the study or outcome level), and how this information is to be used in any data synthesis.	p.7-8<br>eAppendix 3
Summary measures	13	State the principal summary measures (e.g., risk ratio, difference in means). *Also describe the use of additional summary measures assessed, such as treatment rankings and surface under the cumulative ranking curve (SUCRA) values, as well as modified approaches used to present summary findings from meta-analyses.*	p.8-9
Planned methods of analysis	14	Describe the methods of handling data and combining results of studies for each network meta-analysis. This should include, but not be limited to:<br>● *Handling of multi-arm trials;*<br>● *Selection of variance structure;*<br>● *Selection of prior distributions in Bayesian analyses; and*<br>● *Assessment of model fit.*	p.8-9
**Assessment of Inconsistency**	**S2**	Describe the statistical methods used to evaluate the agreement of direct and indirect evidence in the treatment network(s) studied. Describe efforts taken to address its presence when found.	p.8-9<br>eAppendix 4
Risk of bias across studies	15	Specify any assessment of risk of bias that may affect the cumulative evidence (e.g., publication bias, selective reporting within studies).	p.9<br>eAppendix 3
Additional analyses	16	Describe methods of additional analyses if done, indicating which were pre-specified. This may include, but not be limited to, the following:<br>● Sensitivity or subgroup analyses;	p.9<br>eAppendix 6

- Meta-regression analyses;
- *Alternative formulations of the treatment network; and*
- *Use of alternative prior distributions for Bayesian analyses (if applicable).*

RESULTS†

Study selection	17	Give numbers of studies screened, assessed for eligibility, and included in the review, with reasons for exclusions at each stage, ideally with a flow diagram.	p.10<br>eFigure 1
**Presentation of network structure**	**S3**	Provide a network graph of the included studies to enable visualization of the geometry of the treatment network.	Figures 1A, 1B
**Summary of network geometry**	**S4**	Provide a brief overview of characteristics of the treatment network. This may include commentary on the abundance of trials and randomized patients for the different interventions and pairwise comparisons in the network, gaps of evidence in the treatment network, and potential biases reflected by the network structure.	eTable 1
Study characteristics	18	For each study, present characteristics for which data were extracted (e.g., study size, PICOS, follow-up period) and provide the citations.	p.10-11<br>eTable 1
Risk of bias within studies	19	Present data on risk of bias of each study and, if available, any outcome level assessment.	eTable 1<br>eAppendix 3
Results of individual studies	20	For all outcomes considered (benefits or harms), present, for each study: 1) simple summary data for each intervention group, and 2) effect estimates and confidence intervals. *Modified approaches may be needed to deal with information from larger networks.*	Figures 2A, 2B<br>eTable 2, 3
Synthesis of results	21	Present results of each meta-analysis done, including confidence/credible intervals. *In larger networks, authors may focus on comparisons versus a particular comparator (e.g. placebo or standard care), with full findings presented in an appendix. League tables and forest plots may be considered to summarize pairwise comparisons.* If additional summary measures were explored (such as treatment rankings), these should also be presented.	p.10-11<br>Figures 2A, 2B<br>eTable 2, 3
**Exploration for inconsistency**	**S5**	Describe results from investigations of inconsistency. This may include such information as measures of model fit to compare consistency and inconsistency models, *P* values from statistical tests, or summary of inconsistency estimates from different parts of the treatment network.	eAppendix 4
Risk of bias across studies	22	Present results of any assessment of risk of bias across studies for the evidence base being studied.	eTable 1<br>eAppendix 3
Results of additional analyses	23	Give results of additional analyses, if done (e.g., sensitivity or subgroup analyses, meta-regression analyses, *alternative network geometries studied, alternative choice of prior distributions for Bayesian analyses,* and so forth).	eAppendix 6

**DISCUSSION**			
Summary of evidence	24	Summarize the main findings, including the strength of evidence for each main outcome; consider their relevance to key groups (e.g., healthcare providers, users, and policy-makers).	p.12-14
Limitations	25	Discuss limitations at study and outcome level (e.g., risk of bias), and at review level (e.g., incomplete retrieval of identified research, reporting bias). *Comment on the validity of the assumptions, such as transitivity and consistency. Comment on any concerns regarding network geometry (e.g., avoidance of certain comparisons).*	p.14-15
Conclusions	26	Provide a general interpretation of the results in the context of other evidence, and implications for future research.	p. 16
**FUNDING**			
Funding	27	Describe sources of funding for the systematic review and other support (e.g., supply of data); role of funders for the systematic review. This should also include information regarding whether funding has been received from manufacturers of treatments in the network and/or whether some of the authors are content experts with professional conflicts of interest that could affect use of treatments in the network.	p.17

PICOS = population, intervention, comparators, outcomes, study design.
* Text in italics indicate wording specific to reporting of network meta-analyses that has been added to guidance from the PRISMA statement.
† Authors may wish to plan for use of appendices to present all relevant information in full detail for items in this section.

eAppendix 6. Additional Results

Adverse events

Regarding adverse events, it is remarkable that few studies reported adverse events systematically or at all. In total, 15 of the 37 studies made a statement regarding adverse events. From these reports, it is apparent that relatively few adverse events occur in the context of OLP treatment. This suggests that OLP is a safe and mostly side effect free treatment. However, due to inconsistent or unreported adverse events, it is difficult to draw a conclusion.

Certainty of the evidence

The certainty of evidence for the network estimates of both samples was examined by using GRADE. The results for study limitations (within study bias), reporting bias (across-studies bias), indirectness, imprecision, heterogeneity, and incoherence can be found in the supplement (eAppendix 3-4, eFigure 2).

Sensitivity analysis

To investigate the impact of high risk studies, we conducted the analyses including only studies in which the risk of bias was low or moderate. In each sample, one study was high risk of bias and thus excluded and compared to the whole sample. The results in the nonclinical network remained unchanged in principal, solely OLP nasal changed from being marginally significant to insignificant. In the nonclinical sample, cOLP pills moved from being significant to non significant, as only one study with a cOLP pills group remained in the network. Otherwise results and heterogeneity measures remained comparable.

To investigate the impact of including studies with subclinical populations within the clinical sample, we conducted a sensitivity analysis by excluding studies with subclinical samples. In principle, the results remained unchanged with a trend for slightly bigger effect sizes when subclinical studies were excluded (see eFigure 3-6 in the supplement for the results of sensitivity analyses). Surprisingly, heterogeneity increased from I^2 = 26.5% (clinical all) to I^2 = 32.6% (clinical without subclinical).

Furthermore, owing to the great variance of included conditions within each of the two networks, we performed subgroup analysis for two broad areas: pain (i.e., chronic back pain, experimental pain, irritable bowel syndrome, knee ostheoarthritis) and psychological (i.e., depression, fatigue, conditions, well-being, insomnia, test anxiety, sadness, relaxation, stress). The results for the clinical pain network (11 studies) showed comparable results to the ones of the whole network, except the treatment programme changed to being significantly better than NT, whereas OLP- moved to being significantly worse than NT. Interestingly, heterogeneity was reduced from I^2 = 26.5% (clinical all) to I^2 = 0% (clinical pain). Within the nonclinical pain sample (N = 4), results did also change only marginally, with OLP nasal not being significantly better than NT anymore. Heterogeneity as well decreased from I^2 = 66% (nonclinical all) to I^2 = 51.7% (nonclinical pain). Within the psychological subsamples results could in general also be replicated (clinical psychological = 10 and nonclinical psychological = 3 studies), with the exception of DP being bigger in the nonclinical sample and the effect size of OLP- changing from -0.03 to 0.30 in the clinical network. Heterogeneity decreased within the clinical sample from I^2 = 26.5% (clinical all) to I^2 = 0% (clinical psychological) and in the nonclinical network from I^2 = 66% (nonclinical all) to I^2 = 0% (nonclinical psychological). Overall, very few studies were included in the networks of these subgroup-analyses.

eFigure 1. Flowchart

PRISMA 2020 flow diagram for new systematic reviews which included searches of databases, registers and other sources

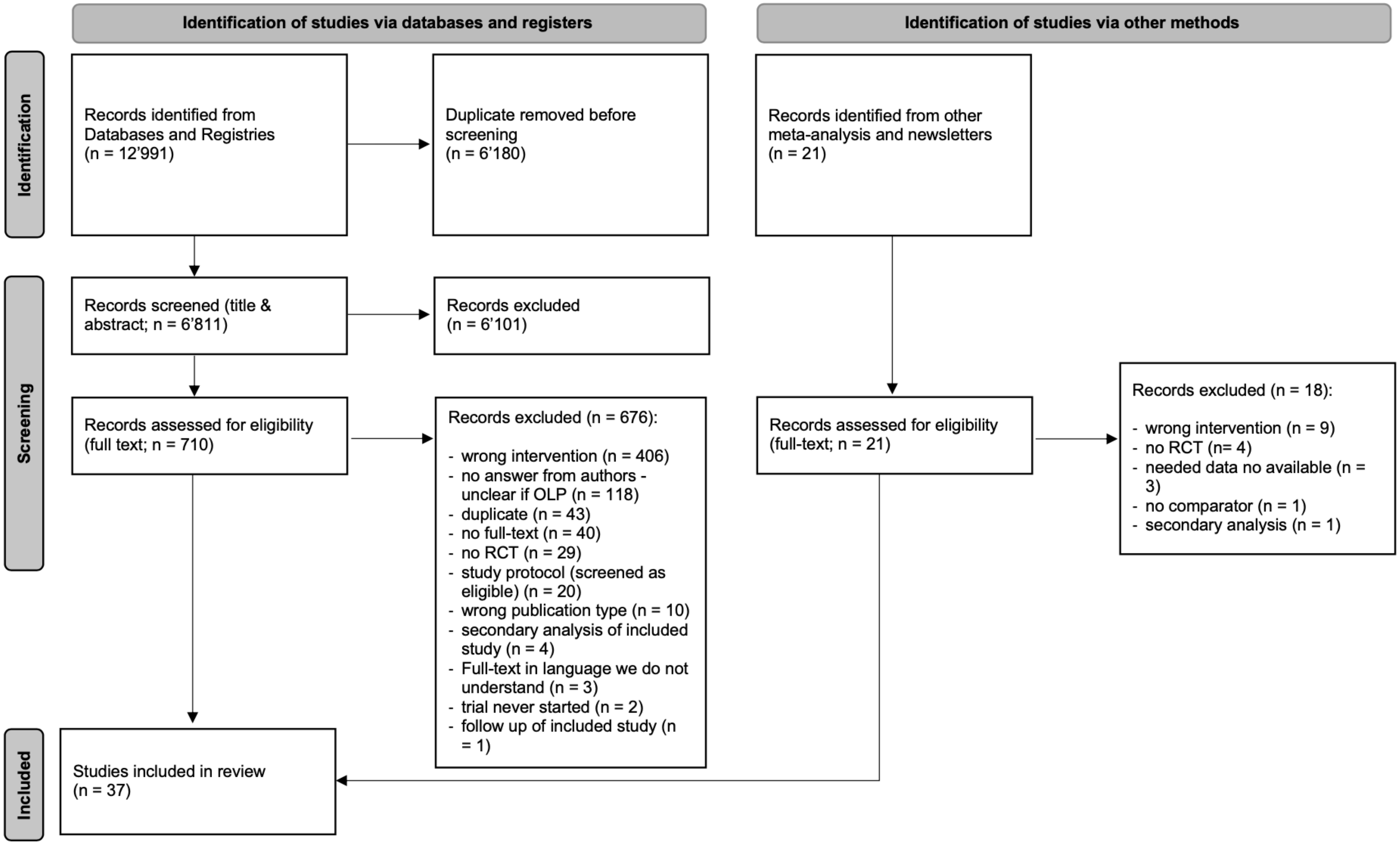

From: Page MJ, McKenzie JE, Bossuyt PM, Boutron I, Hoffmann TC, Mulrow CD, et al. The PRISMA 2020 statement: an updated guideline for reporting systematic reviews. BMJ 2021;372:n71. doi: 10.1136/bmj.n71. For more information, visit: http://www.prisma-statement.org/

eFigure 2. Funnel plots with accompanying Egger test

A. Nonclinical network

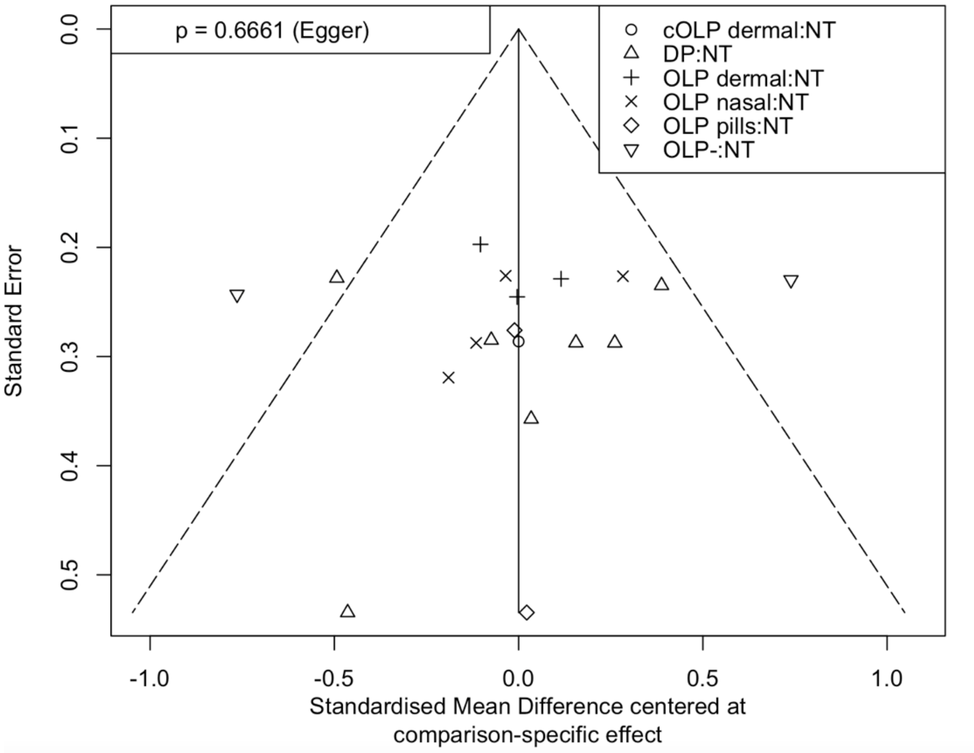

B. Clinical network

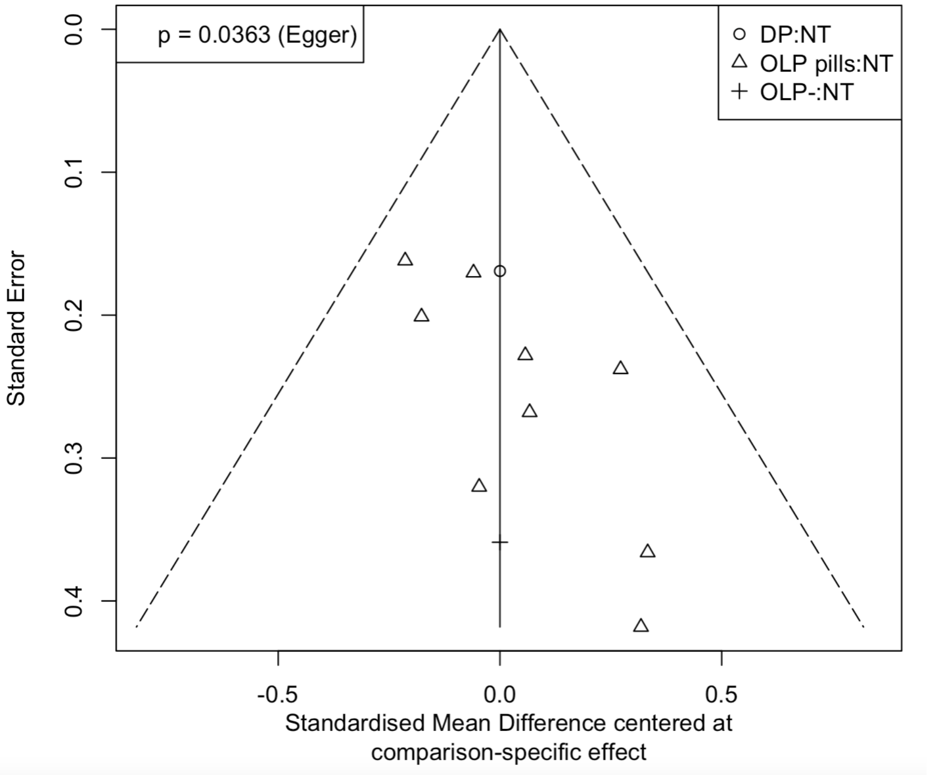

Note: Funnel plot with reference NT, i.e. this plot only includes studies with NT as a control group depicting available comparisons with DP and OLP.

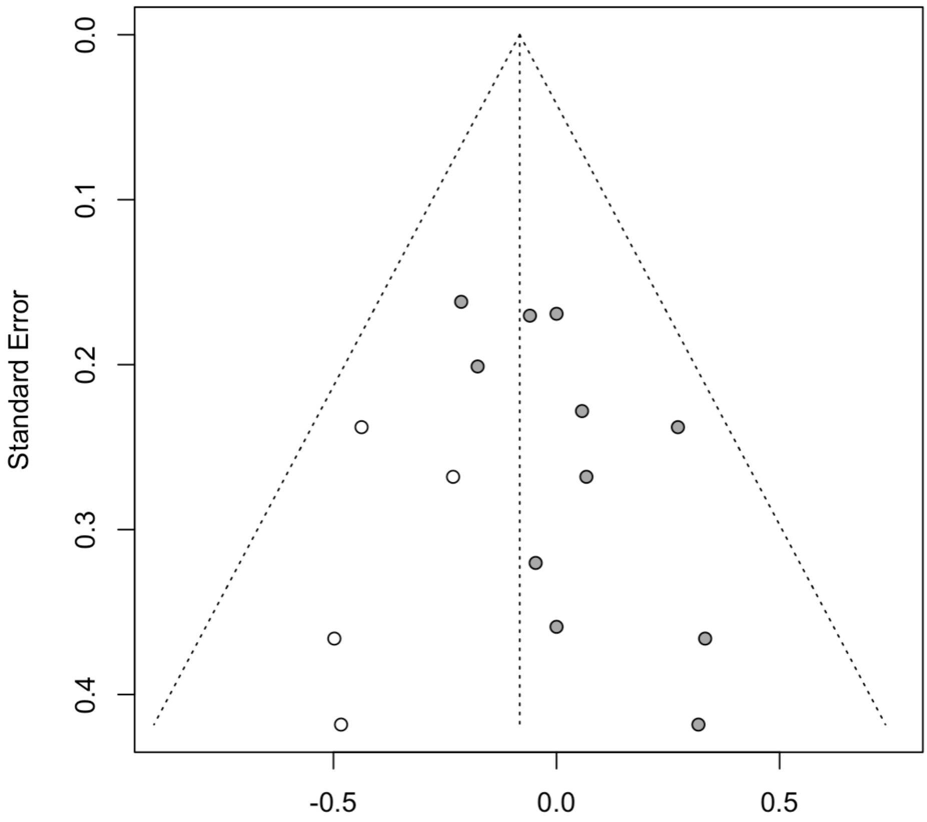

Note: Funnel plot with reference NT after using the trim and fill method. The plot depicts the four comparisons (white dots) NT vs. OLP pills that are missing in order for the funnel to be symmetric.

eFigure 3. Plots of low and moderate risk of bias only (sensitivity analysis)

A. Netgraph of nonclinical network meta-analysis on low and moderate risk of bias only

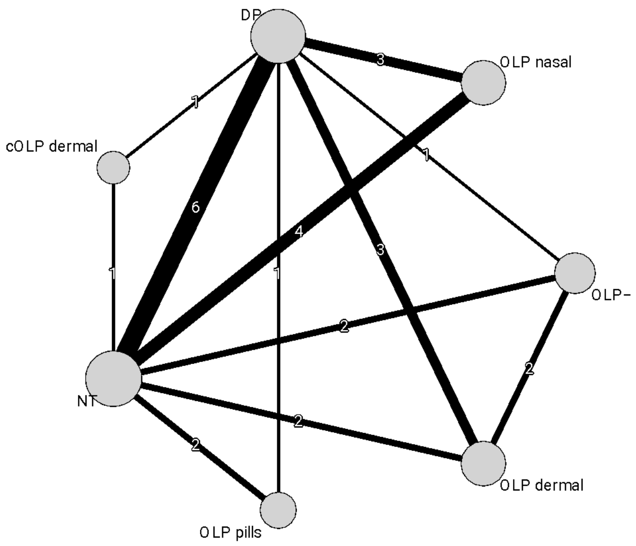

B. Forest plot of nonclinical network meta-analysis on low and moderate risk of bias only

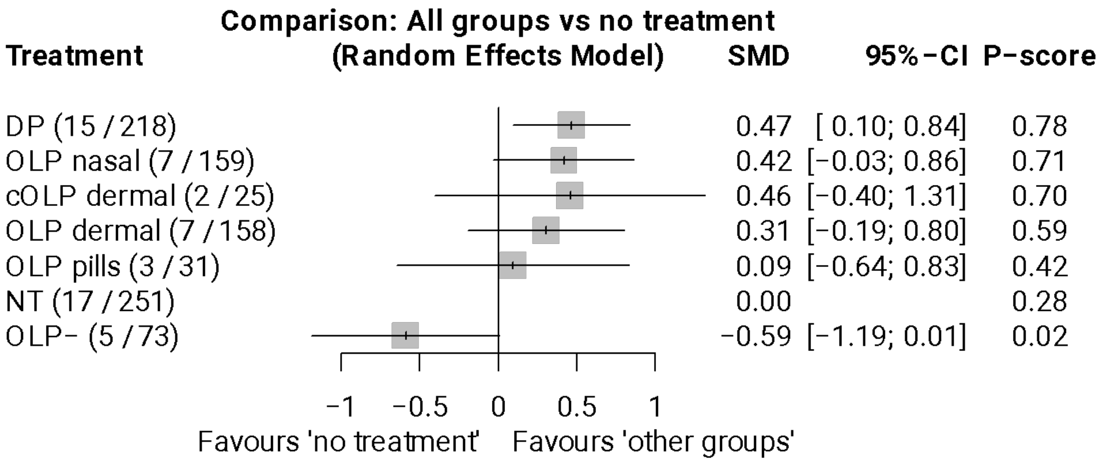

C. **Netgraph of clinical network meta-analysis on low and moderate risk of bias only**

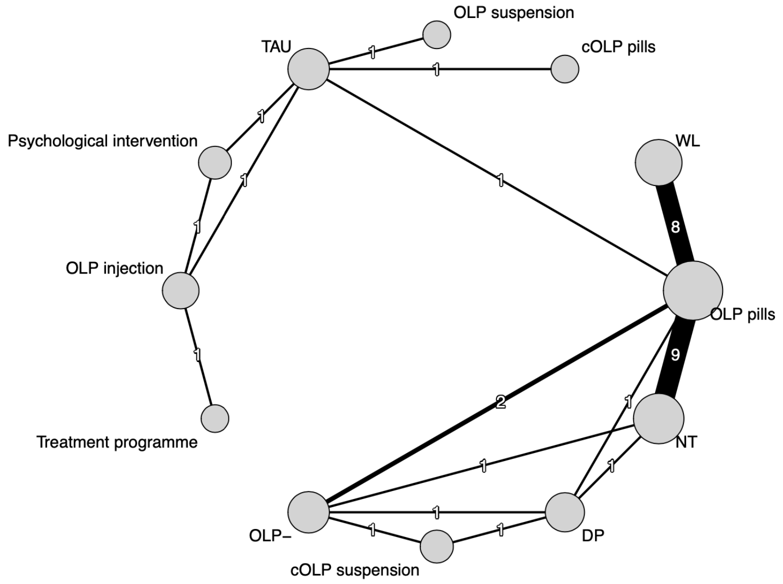

D. **Forest plot of clinical network meta-analysis on low and moderate risk of bias only**

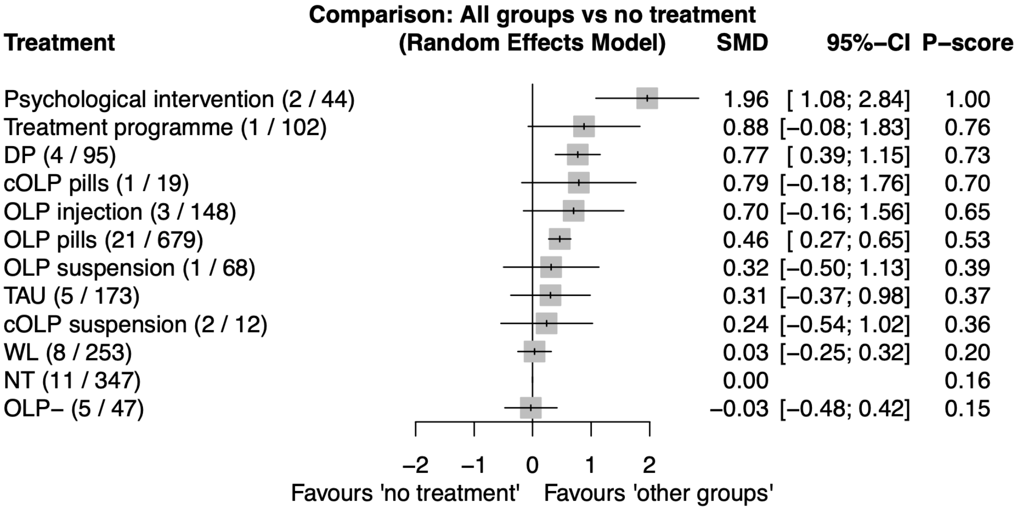

eFigure 4. Plots of clinical network without subclinical trials (sensitivity analysis)

A. Netgraph of network meta-analysis on clinical studies only

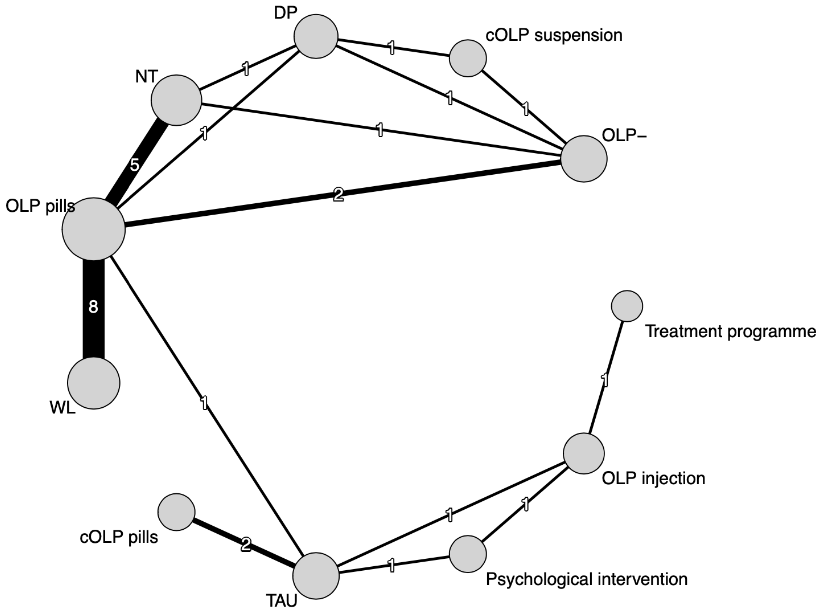

B. Forest plot of network-meta-analysis on clinical studies only

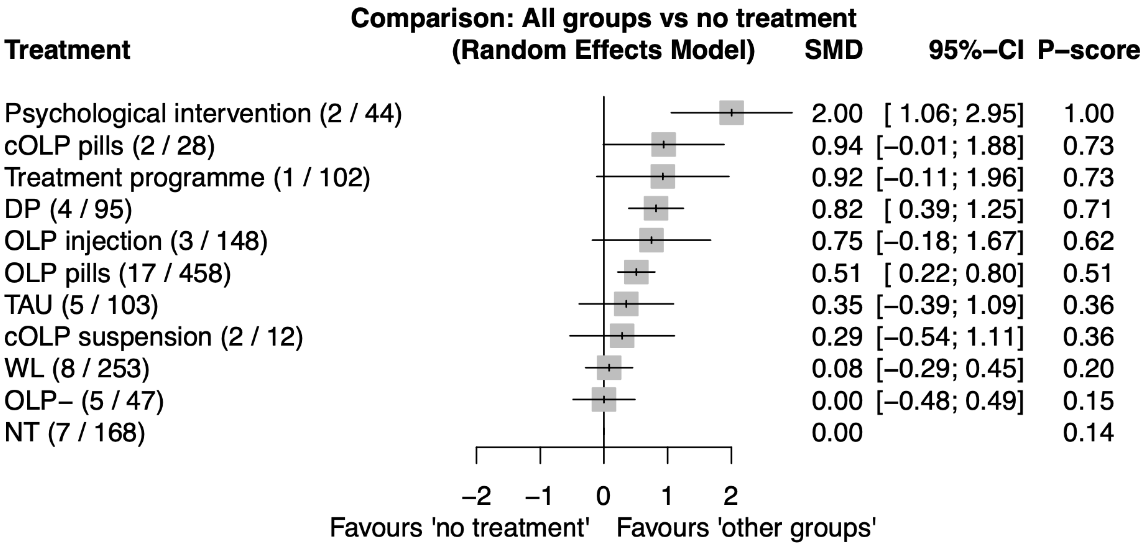

eFigure 5. Plots of pain trials (sensitivity analysis)

A. **Netgraph of network meta-analysis on clinical pain studies only**

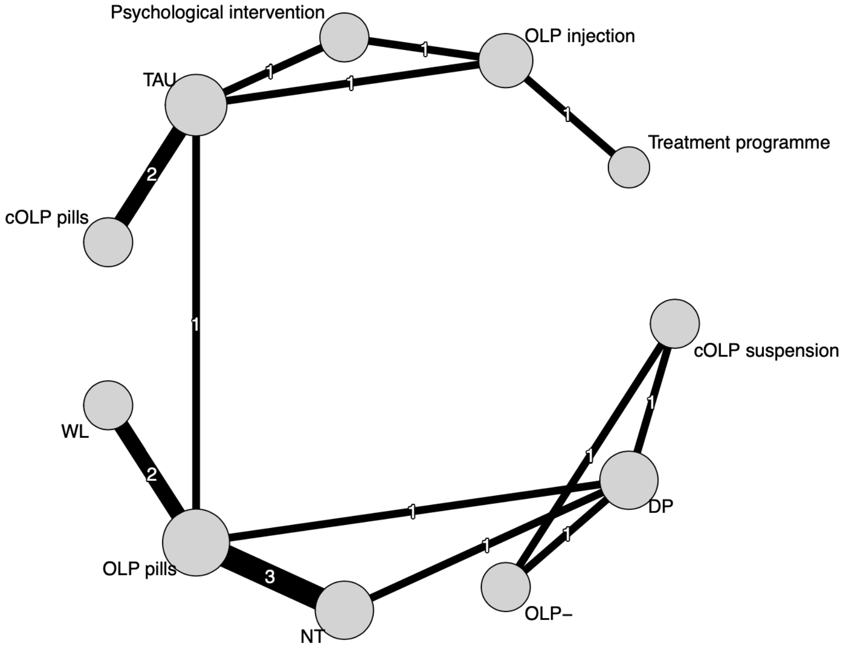

B. **Forest plot of network meta-analysis on clinical pain studies only**

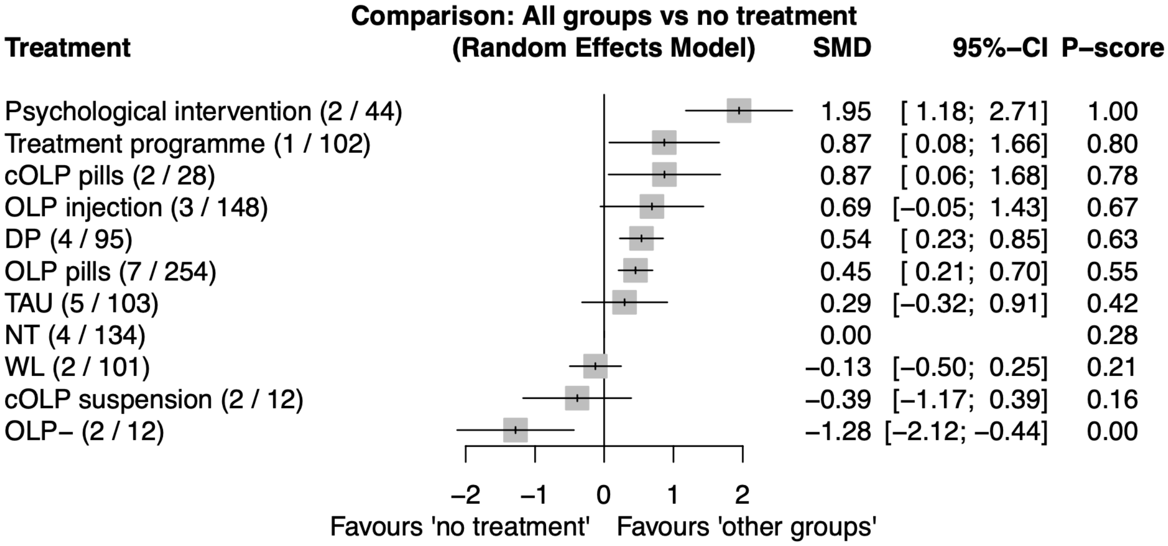

C. Netgraph of network meta-analysis on nonclinical pain studies only

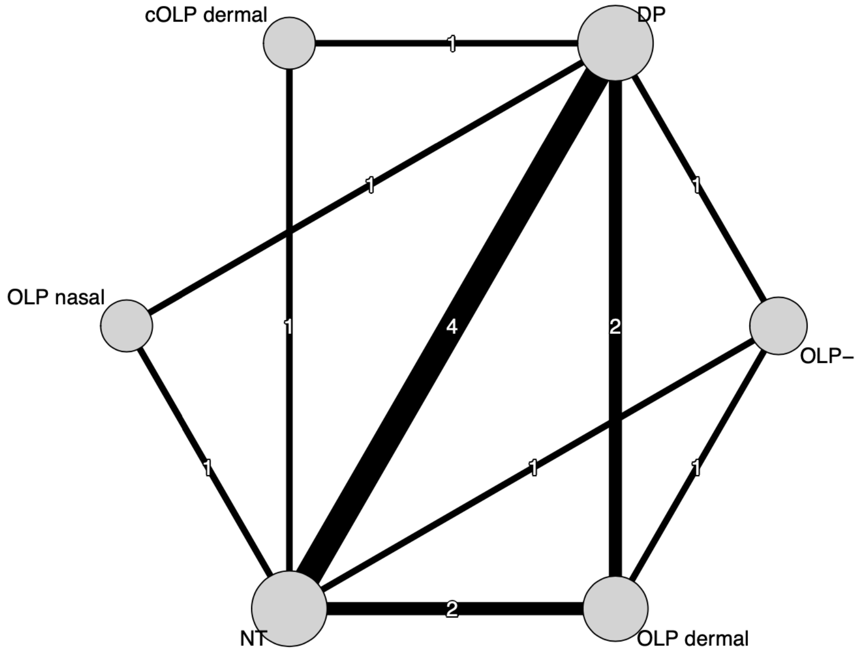

D. Forest plot of network meta-analysis on nonclinical pain studies only

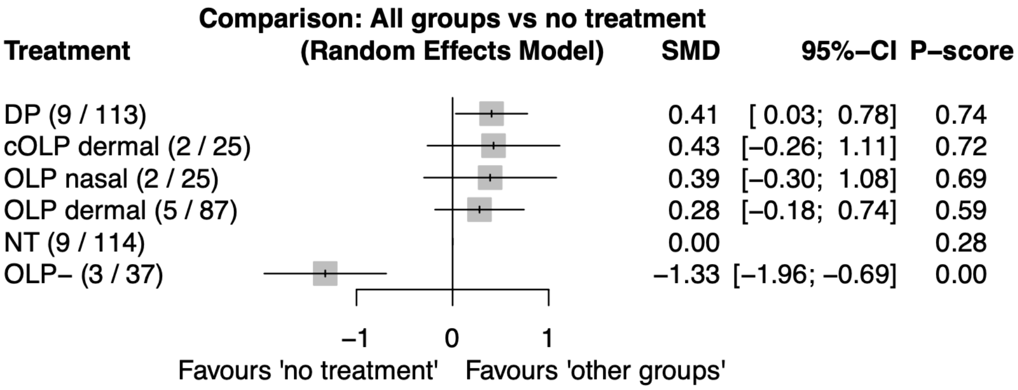

eFigure 6. Plots of psychological trials (sensitivity analysis)

A. **Netgraph of network meta-analysis on clinical psychological studies only**

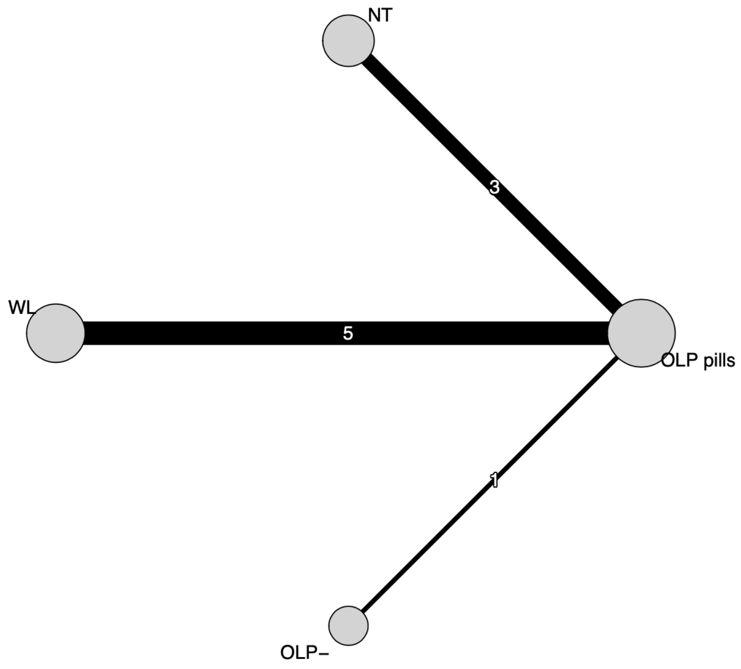

B. **Forest plot of network meta-analysis on clinical psychological studies only**

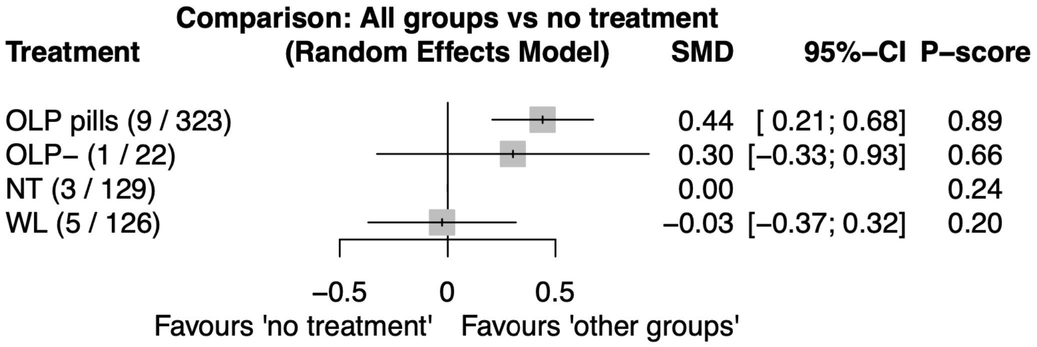

C. **Netgraph of network meta-analysis on nonclinical psychological studies only**

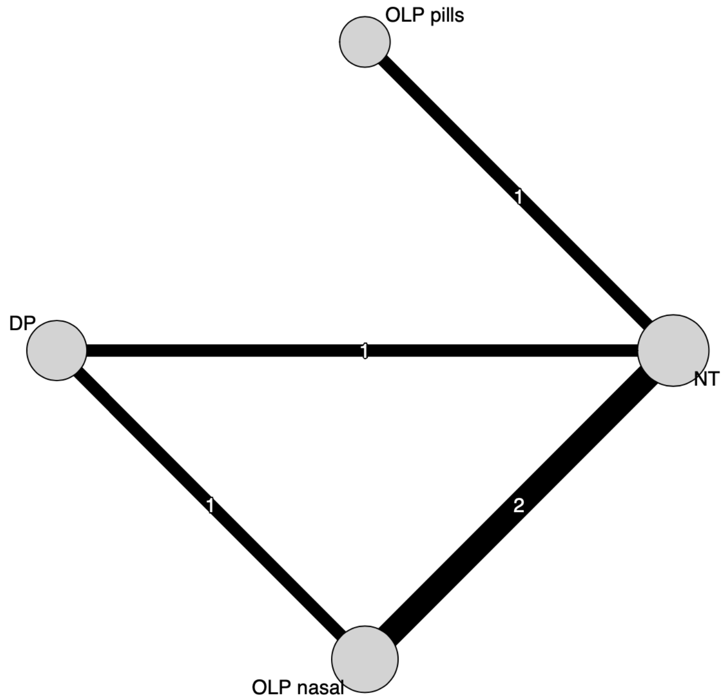

D. **Forest plot of network meta-analysis on nonclinical psychological studies only**

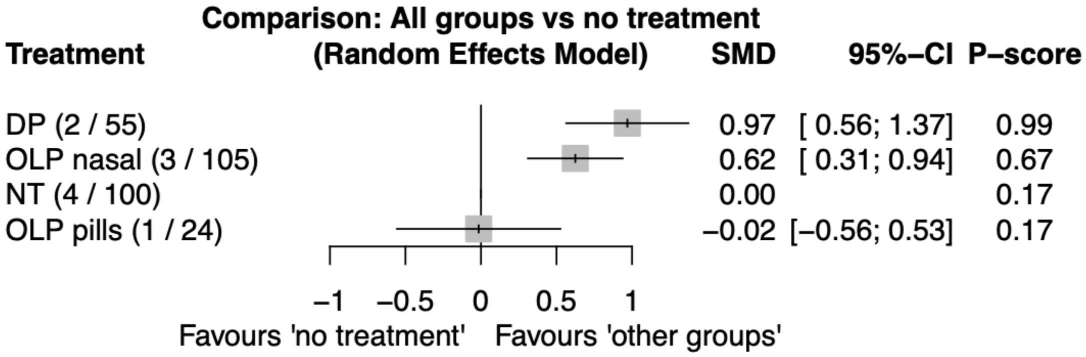

eTable 1. Demographics and study characteristic

Author, Year	Country	Condition /Diagnosis	Sample used in analysis	N (% female) per group	Mean age in years (SD) per group	Treatment duration in days	Intervention 1	Intervention 2	Intervention 3	Control	Outcome used for analysis	Rationale for choice of outcome	Risk of Bias
Ashar, 2021	USA	chronic low back pain	clinical	135 (53.67)	41.10 (15.67)	28	OLP injection (injection)	psychological intervention		TAU	pain intensity (NRS 0-10)	only PO	low
Bandak, 2022	Denmark	knee osteoarthritis	clinical	206 (45.65)	68.40 (8.25)	56	OLP injection (injection)			Treatment program (exercise and education)	pain subscale (KOOS 0-100)	only PO	some concerns
Barnes, 2019	Australia	experimental nausea	nonclinical	61 (52.74)	21.50 (4.65)	2	OLP nasal (vapor) (semi + fully open)	DP (vapor)		NT	nausea (VAS 0-10)	only PO	some concerns
Carvalho, 2016	Portugal	chronic low back pain	clinical	83 (71.05)	44.25 (13.45)	21	OLP pills (pill)			WL	pain intensity (NRS 0-10)	most frequent	some concerns

Author, Year	Country	Condition /Diagnosis	Sample used in analysis	N (% female) per group	Mean age in years (SD) per group	Treatment duration in days	Intervention 1	Intervention 2	Intervention 3	Control	Outcome used for analysis	Rationale for choice of outcome	Risk of Bias
Disley, 2021	UK	experimental pain	nonclinical	75 (86.67)	21.05 (5.04)	1	OLP nasal (spray)	DP (spray)		NT	pain intensity (VAS 0-100)	most frequent	some concerns
El Brihi, 2019	Australia	well-being	subclinical	88 (80.00)	19.00 (3.90)	7	OLP pills (capsule) (different doses merged)			NT	emotional distress (DASS)	most frequent	some concerns
Flowers, 2021	USA	acute pain (following spine surgery)	clinical	41 (NA)	60.15 (13.05)	17	cOLP pills (pill)			TAU	worst daily pain (mini-BP; NRS 0-10)	most frequent	some concerns
Friehs, 2022	Germany	experimental sadness	nonclinical	147 (70.26)	23.56 (4.25)	7	OLP nasal (spray sesame oil) (personal + scientific)	DP (spray)		NT (personal + scientific)	sadness subscale (PANAS-X)	only PO	some concerns
Haas, 2022	Germany	primary insomnia	clinical	45 (84.39)	30.07 (NA)	2	OLP pills (pill)			OLP- (pill)	subjective total sleep time in minutes	only PO	some concerns

**Author, Year**	**Country**	**Condition/Diagnosis**	**Sample used in analysis**	**N (% female) per group**	**Mean age in years (SD) per group**	**Treatment duration in days**	**Intervention 1**	**Intervention 2**	**Intervention 3**	**Control**	**Outcome used for analysis**	**Rationale for choice of outcome**	**Risk of Bias**
Hahn, 2022	Germany	experimental sadness	nonclinical	84 (100.00)	24.74 (5.15)	1	OLP nasal (spray)			NT	sadness subscale (PANAS-X)	only PO	some concerns
Hoenemeyer, 2018	USA	cancer-related fatigue	clinical	73 (69.00)	57.20 (11.80)	21	OLP pills (pill)			WL	cancer related fatigue (FSI-14)	most frequent	some concerns
Ikemoto, 2020	Japan	chronic low back pain	clinical	48 (61.55)	66.75 (66.75)	84	OLP pills (pill)			TAU	pain intensity (NRS 0-10)	most frequent	some concerns
Kaptchuk, 2010	Israel	irritable bowel syndrome	clinical	80 (69.50)	46.50 (18.00)	21	OLP pills (pill)			NT	IBS symptom severity scale (IBS-SSS)	baseline available	some concerns
Kelley, 2012	USA	major depressive disorder	clinical	20 (70.00)	38.80 (12.60)	14	OLP pills (capsule)			WL	depression severity (HAM-D-17)	only PO	some concerns

Author, Year	Country	Condition/Diagnosis	Sample used in analysis	N (% female) per group	Mean age in years (SD) per group	Treatment duration in days	Intervention 1	Intervention 2	Intervention 3	Control	Outcome used for analysis	Rationale for choice of outcome	Risk of Bias
Kleine-Borgmann, 2021	Germany	chronic low back pain	clinical	122 (NA)	59.33 (14.56)	21	OLP pills (capsule)			WL	pain intensity (NRS 0-10)	only PO	some concerns
Kleine-Borgmann, 2019	Germany	well-being & cognitive enhancement	subclinical	154 (67.50)	24.03 (2.79)	21	OLP pills (pill)			NT	stress (PSQ-20)	baseline available; most informative	some concerns
Klinger, 2017	Germany	chronic low back pain + experimental pain	clinical	48 (75.00)	50.89 (15.07)	1	cOLP suspension (saline cotton swab)	OLP-(saline cotton swab)		DP (conditioned + unconditioned), (saline cotton swab)	pain intensity (NRS 0-10)	only PO	some concerns
Kube, 2020	Germany	experimental pain	nonclinical	100 (49.50)	24.56 (5.66)	1	OLP dermal (cream) (expectancy + hope)	DP (cream)		NT	pain intensity (VAS 0-100)	only PO	high

Author, Year	Country	Condition/Diagnosis	Sample used in analysis	N (% female) per group	Mean age in years (SD) per group	Treatment duration in days	Intervention 1	Intervention 2	Intervention 3	Control	Outcome used for analysis	Rationale for choice of outcome	Risk of Bias
Kube, 2021	Germany	allergic rhinitis	clinical	54 (68.68)	31.48 (12.67)	14	OLP pills (tablet) (augmented + limited)			WL (augmented + limited)	self-reported allergic symptoms (CSMS)	only PO	some concerns
Leibowitz, 2019	USA	experimental itch	nonclinical	NA (63.50)	24.55 (NA)	NA	OLP dermal (cream) (expectation + rationale)	OLP- (cream)		NT	physiological allergic reaction (size of the wheal)	only PO	some concerns
Lembo, 2021	USA	irritable bowel syndrome	clinical	211 (72.93)	42.00 (18.00)	42	OLP pills (pill)	DP (pill)		NT	IBS symptom severity scale (IBS-SSS)	only PO	some concerns
Locher, 2017	Switzerland	experimental pain	nonclinical	151 (68.00)	27.15 (9.51)	1	OLP dermal (cream)	OLP- (cream)	DP (cream)	NT	pain intensity (VAS 0-100)	most frequent	low

Author, Year	Country	Condition/Diagnosis	Sample used in analysis	N (% female) per group	Mean age in years (SD) per group	Treatment duration in days	Intervention 1	Intervention 2	Intervention 3	Control	Outcome used for analysis	Rationale for choice of outcome	Risk of Bias
Meeuwis, 2021	Netherlands	experimental itch	nonclinical	55 (85.45)	21.89 (2.50)	1	OLP dermal (patch)			DP (patch)	mean itch (NRS 0-10)	only PO	some concerns
Meeuwis, 2019	Netherlands	experimental itch	nonclinical	45 (82.60)	21.80 (2.70)	7	OLP dermal (tonic)			DP (tonic)	AUC itch (NRS 0-10)	only PO	some concerns
Morales-Quezada, 2020	USA	acute pain (spinal cord injury and polytrauma)	clinical	19 (30.00)	47.30 (16.78)	6	cOLP pills (capsule)			TAU	opioid consumption (MEDC)	only PO	high
Mundt, 2017	USA	experimental pain	nonclinical	75 (57.33)	22.75 (5.89)	1	cOLP dermal (cream)	DP (cream)		NT	pain intensity (VAS 0-100)	only PO	some concerns
Nitzan, 2020	Israel	major depressive disorder	clinical	38 (NA)	49.91 (17.27)	56	OLP pills (capsule)			WL	depression severity (QIDS)	only PO	some concerns

Author, Year	Country	Condition/Diagnosis	Sample used in analysis	N (% female) per group	Mean age in years (SD) per group	Treatment duration in days	Intervention 1	Intervention 2	Intervention 3	Control	Outcome used for analysis	Rationale for choice of outcome	Risk of Bias
Olliges, 2022	Germany	knee osteoarthritis	clinical	40 (60.15)	67.02 (9.47)	21	OLP pills (capsule)			NT	pain intensity (NRS 0-10)	most frequent	some concerns
Pan, 2020	Germany	menopausal hot flushes	subclinical	100 (100.00)	54.55 (NA)	28	OLP pills (pill)			NT	hot flushes composite score	most informative	some concerns
Schaefer, 2018	Germany	allergic rhinitis	clinical	46 (77.80)	24.67 (6.37)	14	OLP pills (pill)	OLP- (pill)		NT (with rationale + without rationale)	allergic symptoms composite score	only PO	some concerns
Schaefer, 2016	Germany	allergic rhinitis	clinical	25 (84.00)	26 (9.90)	14	OLP pills (pill)			NT	allergic symptoms composite score	only PO	some concerns
Schaefer, 2019	Germany	test anxiety	subclinical	58 (86.60)	22.90 (2.85)	14	OLP pills (pill)			NT	test anxiety (PAF)	most informative	some concerns
Schaefer, 2021	Germany	experimental acute stress	nonclinical	53 (53.31)	26.33 (8.77)	21	OLP pills (pill)			NT	acute stress (0-100)	most frequent	some concerns

Author, Year	Country	Condition/Diagnosis	Sample used in analysis	N (% female) per group	Mean age in years (SD) per group	Treatment duration in days	Intervention 1	Intervention 2	Intervention 3	Control	Outcome used for analysis	Rationale for choice of outcome	Risk of Bias
Schienle, 2021	Austria	relaxation	subclinical	148 (71.00)	24.40 (2.70)	14	OLP suspension (sunflower oil)			TAU	PMR exercise quality: relaxation	baseline available; most informative	some concerns
Swafford, 2019*	USA	muscle strength	nonclinical	21 (47.60)	22.52 (3.00)	7	OLP pills (capsule)	DP (capsule)		NT	isometric peak torque	authors judgment	some concerns
Yennurajalingam, 2022	USA	cancer-related fatigue	clinical	84 (67.00)	56.00 (13.00)	7	OLP pills (tablet)			WL	cancer related fatigue (FACIT-F)	only PO	some concerns
Zhou, 2019	USA	cancer-related fatigue	clinical	40 (92.50)	47.30 (12.40)	22	OLP pills (tablet)			WL	cancer related fatigue (FACIT-F)	only PO	some concerns

Note. cOLP, conditioned Open-Label Placebo; DP, Deceptive Placebo; NT, No Treatment; OLP, Open-Label Placebo with rationale; OLP-, Open-Label Placebo without expectation induction; PO, Primary Outcome; TAU, Treatment as Usual; WL, Wait List; *, crossover study

eTable 2. Individual study data

Nonclinical network

author	year	merged groups	data from author	group	population	age mean	age sd	% female	country	continuous outcome	n	mean change	sd change
Barnes	2019	yes (fully & semi open)	yes	OLP nasal	experimental nausea	20.3	3.26	58.62	Australia	Self-report nausea, 6-item composite scale	29	6.14	9.78
Barnes	2019	no	yes	DP	experimental nausea	21.3	5.2	NA	Australia	Self-report nausea, 6-item composite scale	17	8.18	11.36
Barnes	2019	no	yes	NT	experimental nausea	22.9	5.5	NA	Australia	Self-report nausea, 6-item composite scale	15	2.86	11.07
Disley	2021	no	no	OLP nasal	experimental pain	21.05	5.04	86.666	UK	Pain Intensitiy, VAS	25	-0.12	20.35
Disley	2021	no	no	DP	experimental pain	21.05	5.04	86.666	UK	Pain Intensitiy, VAS	26	0.08	21.60
Disley	2021	no	no	NT	experimental pain	21.05	5.04	86.666	UK	Pain Intensitiy, VAS	24	-7.79	17.31
Friehs	2022	yes (personal & scientific)	no	OLP nasal	experimental sadness	24.56	6.55	69.79	Germany	Sadness subscale PANAS-X score total score 0-50	63	-2.20	9.60
Friehs	2022	yes (personal & scientific)	no	DP	experimental sadness	23.02	3.31	58.18	Germany	Sadness subscale PANAS-X score total score 0-50	55	1.00	6.06
Friehs	2022	no	no	NT	experimental sadness	23.1	2.9	82.8	Germany	Sadness subscale PANAS-X score total score 0-50	29	-6.00	8.22
Hahn	2022	no	no	OLP nasal	experimental sadness	23.67	3.31	100	Germany	Sadness subscale PANAS-X score total score 0-50	42	-4.27	8.76
Hahn	2022	no	no	NT	experimental sadness	25.81	6.98	100	Germany	Sadness subscale PANAS-X score total score 0-50	42	-12.01	10.87
Kube	2020	yes (Expectancy & Hope)	no	OLP dermal	experimental pain	25.16	6.41	62	Germany	Pain Intensitiy, VAS	50	-0.02	13.63

Kube	2020	no	no	DP	experimental pain	23.6	4.81	48	Germany	Pain Intensitiy, VAS	25	7.29	13.48
Kube	2020	no	no	NT	experimental pain	24.92	5.76	38.5	Germany	Pain Intensitiy, VAS	25	-2.70	13.92
Leibowitz	2019	no	yes	NT	experimental itch	24.55	NA	63.5	USA	Physiological allergic reaction (size of the wheal)	40	-1.65	1.09
Leibowitz	2019	no	yes	OLP-	experimental itch	24.55	NA	63.5	USA	Physiological allergic reaction (size of the wheal)	36	-1.61	0.83
Leibowitz	2019	yes (expectation & rationale)	yes	OLP dermal	experimental itch	24.55	NA	63.5	USA	Physiological allergic reaction (size of the wheal)	72	-1.56	0.88
Locher	2017	no	no	NT	experimental pain	27.9	8.52	73	Switzerland	Subjective heat pain intensity	40	1.89	3.33
Locher	2017	no	no	OLP-	experimental pain	28.27	11.34	65	Switzerland	Subjective heat pain intensity	37	-3.11	3.46
Locher	2017	no	no	OLP dermal	experimental pain	25.7	7.76	73	Switzerland	Subjective heat pain intensity	37	2.97	3.46
Locher	2017	no	no	DP	experimental pain	26.65	10.25	62	Switzerland	Subjective heat pain intensity	37	1.81	3.46
Meeuwis	2021	no	no	OLP dermal	experimental itch	21.67	2.6	85.19	Netherlands	Self reported mean itch, NRS	27	0.55	1.53
Meeuwis	2021	no	no	DP	experimental itch	22.11	2.39	85.71	Netherlands	Self reported mean itch, NRS	28	0.81	1.48
Meeuwis	2019	no	no	OLP dermal	experimental itch	21.8	2.7	82.6	Netherlands	AUC itch	22	49.71	223.04
Meeuwis	2019	no	no	DP	experimental itch	21.8	2.7	82.6	Netherlands	AUC itch	23	58.27	259.11
Mundt	2017	no	no	NT	experimental pain	22.75	5.89	57.33	USA	Mean pain intensity ratings, VAS	25	-6.14	11.33
Mundt	2017	no	no	DP	experimental pain	22.75	5.89	57.33	USA	Mean pain intensity ratings, VAS	25	1.25	12.46
Mundt	2017	no	no	cOLP dermal	experimental pain	22.75	5.89	57.33	USA	Mean pain intensity ratings, VAS	25	0.21	11.64
Schaefer	2021	no	no	OLP pills	experimental acute stress	25.25	7.28	58.33	Germany	Perceived stress, VAS	24	-31.29	35.07

Schaefer	2021	no	no	NT	experimental acute stress	27.41	10.25	48.28	Germany	Perceived stress, VAS	29	-30.76	34.60
Swafford	2019	no	yes	DP	muscle strength	22.52	3	47.6	USA	Peak torque of experiment 1	7	5.20	55.11
Swafford	2019	no	yes	OLP pills	muscle strength	22.52	3	47.6	USA	Peak torque of experiment 1	7	5.80	58.98
Swafford	2019	no	yes	NT	muscle strength	22.52	3	47.6	USA	Peak torque of experiment 1	7	4.90	32.06

Clinical network

**author**	**year**	**merged groups**	**data from author**	**group**	**population**	**age mean**	**age sd**	**% female**	**country**	**continuous outcome**	**n**	**mean change**	**sd change**
Ashar	2021	no	no	Psycho-logical intervention	chronic low back pain	42.6	16.2	58	USA	Pain intensity, VAS	44	3.04	1.23
Ashar	2021	no	no	OLP injection	chronic low back pain	39.4	14.9	49	USA	Pain intensity, VAS	44	1.32	1.51
Ashar	2021	no	no	TAU	chronic low back pain	41.3	15.9	54	USA	Pain intensity, VAS	47	0.78	1.36
Bandak	2022	no	no	OLP injection	knee osteoarthritis	66.7	8.2	47.2	Denmark	Pain score, KOOS (baseline-week 9)	104	7.30	15.22
Bandak	2022	no	no	Treatment program	knee osteoarthritis	70.1	8.3	44.1	Denmark	Pain score, KOOS (baseline-week 9)	102	10.00	15.07
Carvalho	2016	no	no	OLP pills	chronic low back pain	44.4	13.2	70.7	Portugal	Pain intensity, NRS	41	1.49	1.68
Carvalho	2016	no	no	WL	chronic low back pain	44.1	13.7	71.4	Portugal	Pain intensity, NRS	42	0.24	1.61
El Brihi	2019	yes (OLP 1/d & 4/d)	yes	OLP pills	well-being	19	3.9	80	Australia	Emotional distress (DASS)	61	7.30	9.16
El Brihi	2019	no	yes	NT	well-being	19	3.9	80	Australia	Emotional distress (DASS)	27	0.20	10.26
Flowers	2021	no	no	cOLP pills	acute pain (after spine surgery)	59.1	13.1	NA	USA	Worst daily pain (mini-BP; 0-10)	19	-0.60	2.26
Flowers	2021	no	no	TAU	acute pain (after spine surgery)	61.2	13	NA	USA	Worst daily pain (mini-BP; 0-10)	22	-1.50	1.44

Haas	2022	no	no	OLP pills	primary insomnia	31.04	NA	86.96	Germany	Subjective total sleep time in minutes	23	24.83	91.13
Haas	2022	no	no	OLP-	primary insomnia	29.09	NA	81.82	Germany	Subjective total sleep time in minutes	22	11.31	104.21
Hoenemeyer	2018	no	no	OLP pills	cancer-related fatigue	58.4	11.2	72	USA	FSI, Fatique Symptom Severity)	38	18.60	23.01
Hoenemeyer	2018	no	no	WL	cancer-related fatigue	56	12.4	66	USA	FSI, Fatique Symptom Severity)	35	6.10	22.75
Ikemoto	2020	no	no	OLP pills	chronic low back pain	68.2	68.2	65.4	Japan	Pain intensity, NRS	24	1.10	1.90
Ikemoto	2020	no	no	TAU	chronic low back pain	65.3	65.3	57.7	Japan	Pain intensity, NRS	24	0.80	1.90
Kaptchuk	2010	no	no	OLP pills	irritable bowel syndrome	47	18	65	Israel	IBS-SSS 0-500	37	92.00	99.00
Kaptchuk	2010	no	no	NT	irritable bowel syndrome	46	18	74	Israel	IBS-SSS 0-500	43	46.00	74.00
Kelley	2012	no	no	OLP pills	MDD	38.8	12.6	70	USA	Depression severity, HAM-D	11	1.64	4.52
Kelley	2012	no	no	WL	MDD	38.8	12.6	70	USA	Depression severity, HAM-D	9	-0.67	4.00
Kleine-Borgmann	2021	no	no	OLP pills	chronic low back pain	60.28	15.15	NA	Germany	Composite pain intensity score	63	0.62	1.81
Kleine-Borgmann	2021	no	no	WL	chronic low back pain	58.37	13.97	NA	Germany	Composite pain intensity score	59	-0.11	1.29
Kleine-Borgmann	2019	no	no	OLP pills	well-being & cognitive enhancement	23.97	2.83	68	Germany	Perceived Stress Questionnaire, PSQ20	79	-11.90	19.67
Kleine-Borgmann	2019	no	no	NT	well-being & cognitive enhancement	24.08	2.74	67	Germany	Perceived Stress Questionnaire, PSQ20	75	-16.74	17.22
Klinger	2017	no	no	OLP-	chronic low back pain + experimental pain	50.83	17.01	75	Germany	Back pain rating, NRS	12	-1.16	1.83

Klinger	2017	no	no	cOLP suspension	chronic low back pain + experimental pain	50.33	15.17	75	Germany	Back pain rating, NRS	12	0.67	2.12
Klinger	2017	yes (cond. & uncond. DP)	no	DP	chronic low back pain + experimental pain	51.52	13.05	75	Germany	Back pain rating, NRS	24	2.58	2.12
Kube	2021	yes (augmente d & limited)	no	OLP pills	allergic rhinitis	26.95	10.56	64.3	Germany	Self-reported allergic symptoms, CSMS	28	2.20	3.81
Kube	2021	yes (augmente d & limited)	no	WL	allergic rhinitis	36	14.77	73.05	Germany	Self-reported allergic symptoms, CSMS	26	2.90	3.76
Lembo	2021	no	no	OLP pills	irritable bowel syndrome	42.2	17.8	71.9	USA	IBS-SSS 0-500	68	90.60	89.50
Lembo	2021	no	no	NT	irritable bowel syndrome	40	17	73.3	USA	IBS-SSS 0-500	72	52.30	87.00
Lembo	2021	no	no	DP	irritable bowel syndrome	43.8	19.2	73.6	USA	IBS-SSS 0-500	71	100.30	99.60
Morales-Quezada	2020	no	yes	cOLP pills	acute pain (spinal cord injury and polytrauma)	44.9	16.93	30	USA	Opiod consumption, MDEC	9	66.00	99.55
Morales-Quezada	2020	no	yes	TAU	acute pain (spinal cord injury and polytrauma)	49.7	16.62	30	USA	Opiod consumption, MDEC	10	3.76	56.51
Nitzan	2020	no	no	OLP pills	major depressive disorder	48.17	16.86	NA	Israel	Depression severity,QIDS total score	18	1.95	5.06
Nitzan	2020	no	no	WL	major depressive disorder	51.65	17.68	NA	Israel	Depression severity, QIDS total score	20	0.45	4.12
Olliges	2022	no	yes	OLP pills	knee osteoarthritis	64.19	9.3	57.1	Germany	Pain intensity, NRS	21	0.44	1.35
Olliges	2022	no	yes	NT	knee osteoarthritis	69.84	9.63	63.2	Germany	Pain intensity, NRS	19	-0.28	1.99
Pan	2020	no	no	OLP pills	menopausal hot flushes	54.2	NA	100	Germany	Hot flush score, composite score	50	6.02	9.71

Pan	2020	no	no	NT	menopausal hot flushes	54.9	NA	100	Germany	Hot flush score, composite score	50	3.26	8.79
Schaefer	2018	no	no	OLP pills	allergic rhinitis	25	9	69.2	Germany	Allergic symptoms composite score	13	0.78	0.67
Schaefer	2018	no	no	OLP-	allergic rhinitis	23	3	69.2	Germany	Allergic symptoms composite score	13	0.43	0.90
Schaefer	2018	yes (with & without rationale)	no	NT	allergic rhinitis	26	7.11	95	Germany	Allergic symptoms composite score	20	0.05	1.03
Schaefer	2016	no	no	OLP pills	allergic rhinitis	26	9.9	84	Germany	Allergic symptoms composite score	11	0.88	0.93
Schaefer	2016	no	no	NT	allergic rhinitis	26	9.9	84	Germany	Allergic symptoms composite score	14	0.23	0.72
Schaefer	2019	no	no	OLP pills	test anxiety	22.3	2.3	80.6	Germany	Test anxiety, PAF	31	4.39	9.35
Schaefer	2019	no	no	NT	test anxiety	23.5	3.4	92.6	Germany	Test anxiety, PAF	27	0.07	6.00
Schienle	2021	no	no	OLP suspension	relaxation	24.4	2.7	71	Austria	Exercise quality relaxation	68	1.29	0.97
Schienle	2021	no	no	TAU	relaxation	24.4	2.7	71	Austria	Exercise quality relaxation	80	1.28	0.93
Yennurajal ingam	2022	no	no	OLP pills	cancer-related fatigue	57	12	74	USA	Fatigue, FACIT-F	42	6.60	7.60
Yennurajal ingam	2022	no	no	WL	cancer-related fatigue	55	14	60	USA	Fatigue, FACIT-F	42	2.10	9.40
Zhou	2019	no	no	OLP pills	cancer-related fatigue	47.3	12.4	92.5	USA	Fatigue, FACIT-F	20	4.30	10.43
Zhou	2019	no	no	WL	cancer-related fatigue	47.3	12.4	92.5	USA	Fatigue, FACIT-F	20	1.20	10.15

Note. cOLP, conditioned Open-Label Placebo; DP, Deceptive Placebo; NT, No Treatment; OLP, Open-Label Placebo with rationale; OLP-, Open-Label Placebo without expectation induction; TAU, Treatment as Usual; WL, Wait List.

eTable 3. Head to head comparisons

Nonclinical network

	**cOLP dermal**	**DP**	**NT**	**OLP dermal**	**OLP nasal**	**OLP pills**	**OLP-**
**cOLP dermal**		-0.09 [-1.00; 0.82]	0.54 [-0.37; 1.45]	.	.	.	.
**DP**	-0.03 [-0.83; 0.78]		**0.47 [0.12; 0.82]**	0.10 [-0.34; 0.54]	0.21 [-0.29; 0.72]	-0.01 [-1.28; 1.26]	**1.44 [0.57; 2.30]**
**NT**	0.47 [-0.33; 1.28]°	**0.50 [0.17; 0.83]**		-0.20 [-0.69; 0.29]	**-0.50 [-0.94; -0.06]**	0.00 [-0.73; 0.74]	**0.70 [0.09; 1.30]**
**OLP dermal**	0.21 [-0.66; 1.08]⁺	0.24 [-0.15; 0.62]	-0.26 [-0.67; 0.14]°		.	.	**0.86 [0.26; 1.46]**
**OLP nasal**	0.04 [-0.83; 0.92]⁺	0.07 [-0.36; 0.50]	**-0.43 [-0.84; -0.02]°**	-0.17 [-0.70; 0.37]⁺		.	.
**OLP pills**	0.38 [-0.68; 1.43]⁺	0.40 [-0.34; 1.15]	-0.10 [-0.80; 0.60]°	0.17 [-0.63; 0.96]⁺	0.33 [-0.47; 1.13]⁺		.
**OLP-**	**1.07 [0.12; 2.02]***	**1.10 [0.53; 1.66]**	**0.60 [0.05; 1.15]**	**0.86 [0.31; 1.41]***	**1.03 [0.37; 1.69]***	0.69 [-0.19; 1.57]*	

Note. Column headers are identical to row headers. Cells contain the network estimates (SMDs) from network meta-analysis (direct and indirect evidence) in the lower triangle and the direct treatment estimates (SMDs) from pairwise comparisons in the upper triangle. Comparisons considered for RQ1 (expectation) are marked with a *, for RQ2 (comparator) marked with a °, and for RQ3 (modalities) marked with a ⁺. Legend: cOLP, conditioned Open-Label Placebo; DP, Deceptive Placebo; NT, No Treatment; OLP, Open-Label Placebo with rationale; OLP-, Open-Label Placebo without expectation induction; TAU, Treatment as Usual; WL, Wait List.

Clinical network

	cOLP pills	cOLP suspension	DP	NT	OLP injection	OLP pills	OLP suspension	OLP-	Psych. intervent.	TAU	Treatment programme	WL
**cOLP pills**		.	.	.	.	.	.	.	.	**0.58 [0.02; 1.15]**	.	.
**cOLP suspension**	0.65 [-0.50; 1.81]$^{+}$		**-0.93 [-1.71; -0.15]**	.	.	.	.	**0.89 [0.02; 1.76]**	.	.	.	.
**DP**	0.12 [-0.81; 1.06]	-0.53 [-1.26; 0.20]		**0.52 [0.07; 0.97]**	.	0.11 [-0.34; 0.55]	.	**1.82 [0.98; 2.66]**	.	.	.	.
**NT**	**0.89 [0.01; 1.76]°**	0.23 [-0.54; 1.01]	**0.76 [0.39; 1.14]**		.	**-0.47 [-0.66; -0.29]**	.	-0.42 [-1.19; 0.34]	.	.	.	.
**OLP injection**	0.19 [-0.57; 0.95]$^{+}$	-0.47 [-1.59; 0.66]$^{+}$	0.06 [-0.84; 0.96]	-0.70 [-1.54; 0.14]		.	.	.	**-1.26 [-1.79; -0.72]**	0.39 [-0.12; 0.91]	-0.18 [-0.58; 0.23]	.
**OLP pills**	0.42 [-0.43; 1.28]$^{+}$	-0.23 [-1.00; 0.54]$^{+}$	0.30 [-0.07; 0.67]	**-0.46 [-0.65; -0.28]°**	0.24 [-0.58; 1.06]$^{+}$		.	0.23 [-0.28; 0.75]	.	0.16 [-0.48; 0.80]	.	**0.43 [0.22; 0.64]**
**OLP suspension**	0.57 [-0.15; 1.29]$^{+}$	-0.08 [-1.18; 1.01]$^{+}$	0.45 [-0.41; 1.31]	-0.32 [-1.12; 0.49]	0.38 [-0.29; 1.06]$^{+}$	0.15 [-0.63; 0.93]$^{+}$		.	.	0.01 [-0.43; 0.45]	.	.
**OLP-**	0.92 [-0.04; 1.87]*	0.26 [-0.51; 1.03]*	**0.79 [0.30; 1.29]**	0.03 [-0.41; 0.47]	0.73 [-0.20; 1.66]*	**0.49 [0.07; 0.92]***	0.35 [-0.54; 1.23]*		.	.	.	.
**Psych. intervent.**	**-1.07 [-1.85; -0.28]**	**-1.72 [-2.87; -0.58]**	**-1.19 [-2.11; -0.27]**	**-1.96 [-2.82; -1.09]**	**-1.26 [-1.79; -0.72]**	**-1.49 [-2.34; -0.65]**	**-1.64 [-2.34; -0.94]**	**-1.99 [-2.93; -1.04]**		**1.65 [1.11; 2.20]**	.	.
**TAU**	**0.58 [0.02; 1.15]°**	-0.07 [-1.08; 0.93]	0.46 [-0.28; 1.20]	-0.30 [-0.97; 0.36]	0.39 [-0.12; 0.91]	0.16 [-0.48; 0.80]°	0.01 [-0.43; 0.45]	-0.34 [-1.11; 0.44]	**1.65 [1.11; 2.20]**		.	.
**Treatment programme**	0.01 [-0.85; 0.87]	-0.65 [-1.84; 0.55]	-0.11 [-1.10; 0.87]	-0.88 [-1.81; 0.06]	-0.18 [-0.58; 0.23]	-0.41 [-1.33; 0.50]	-0.56 [-1.35; 0.23]	-0.91 [-1.92; 0.10]	**1.08 [0.41; 1.75]**	-0.57 [-1.23; 0.08]		.
**WL**	0.86 [-0.02; 1.74]°	0.20 [-0.60; 1.00]	**0.73 [0.31; 1.16]**	-0.03 [-0.31; 0.25]	0.67 [-0.18; 1.51]	**0.43 [0.22; 0.64]°**	0.28 [-0.52; 1.09]	-0.06 [-0.54; 0.41]	**1.92 [1.06; 2.79]**	0.27 [-0.40; 0.95]	0.85 [-0.09; 1.78]	

Note. Column headers are identical to row headers. Cells contain the network estimates (SMDs) from network meta-analysis (direct and indirect evidence) in the lower triangle and the direct treatment estimates (SMDs) from pairwise comparisons in the upper triangle. Comparisons considered for RQ1 (expectation) are marked with a *, for RQ2 (comparator) marked with a °, for RQ3 (modalities) marked with a $^{+}$. Legend: cOLP, conditioned Open-Label Placebo; DP, Deceptive Placebo; NT, No Treatment; OLP, Open-Label Placebo with rationale; OLP-, Open-Label Placebo without expectation induction; TAU, Treatment as Usual; WL, Wait List.

eReferences

1. Salanti, G., Del Giovane, C., Chaimani, A., Caldwell, D. M. & Higgins, J. P. Evaluating the quality of evidence from a network meta-analysis. *PloS One* **9**, e99682 (2014).
2. Papakonstantinou, T., Nikolakopoulou, A., Higgins, J. P., Egger, M. & Salanti, G. Cinema: software for semiautomated assessment of the confidence in the results of network meta-analysis. *Campbell Syst. Rev.* **16**, e1080 (2020).
3. Nikolakopoulou, A. *et al.* CINeMA: an approach for assessing confidence in the results of a network meta-analysis. *PLoS Med.* **17**, e1003082 (2020).
4. Sterne, J. A. C. *et al.* RoB 2: a revised tool for assessing risk of bias in randomised trials. *Bmj* **366**, l4898 (2019).
5. Chiocchia, V. *et al.* ROB-MEN: a tool to assess risk of bias due to missing evidence in network meta-analysis. *BMC Med.* **19**, 1–13 (2021).
6. Cuijpers, P., Noma, H., Karyotaki, E., Cipriani, A. & Furukawa, T. A. Effectiveness and acceptability of cognitive behavior therapy delivery formats in adults with depression: a network meta-analysis. *JAMA Psychiatry* **76**, 70047–707 (2019).
